# Synergistic nanoparticle dyad improving tumor immunogenicity and reshaping tolerogenic dendritic cells in hepatocellular carcinoma

**DOI:** 10.1016/j.mtbio.2026.103488

**Published:** 2026-07-23

**Authors:** Chang Liu, Xiejun Zhao, Hui Song, Yiwen Yuan, Jia Ma, Yingtong Dong, Tianzhao Xu, Xiaojiao Li, Xinghui Liu

**Affiliations:** a Department of Clinical Laboratory, Pudong Gongli Hospital, Shanghai University of Medicine&Health Sciences, Shanghai, PR China; b School of Pharmacy, Health Science Center, Xi'an Jiaotong University, Xi'an, Shaanxi, PR China; c BioBank, The First Affiliated Hospital of Xi'an Jiaotong University, Xi'an, Shaanxi, 710061, PR China; d Department of Scientific Research, Pudong Zhoupu Hospital, Shanghai University of Medicine & Health Sciences, Shanghai, PR China

**Keywords:** Hepatocellular carcinoma, Nanoparticle dyad, Tumor immunogenicity, Dendritic cell activation

## Abstract

Hepatocellular carcinoma (HCC) remains a global health challenge with limited treatment efficacy despite advances in immune checkpoint blockade (ICB). The low immunogenicity of HCC tumors and impaired dendritic cell (DC) function contribute to suboptimal therapeutic outcomes. To address these limitations, we developed a synergistic nanoparticle dyad combining tumoricidal and immunomodulatory components. Specifically, an anti-GPC3-antibody-conjugated, emodin-loaded spiky mesoporous silica nanoparticle (E-SMSNα) selectively targets HCC cells to induce immunogenic cell death, while a matured DC-membrane-camouflaged, c-di-AMP-loaded nanoparticle (A-MSNm) reprograms dysfunctional DCs, bridging innate and adaptive immunity. This combinatorial approach synergistically promotes antitumor immunity, significantly suppressing primary tumor growth and inducing durable protection against tumor rechallenge. Moreover, it shows strong synergy with PD-1 checkpoint blockade, positioning it as a promising strategy for HCC immunotherapy.

## Highlights

• A synergistic nanoparticle dyad (E-SMSNα + A-MSNm) was developed to simultaneously enhance tumor immunogenicity and reshape tolerogenic dendritic cells in hepatocellular carcinoma.
• Anti-GPC3-conjugated spiky mesoporous silica nanoparticles (E-SMSNα) enable tumor-targeted emodin delivery, efficiently inducing cancer cell apoptosis and tumor-associated antigen release.
• The nanoparticle dyad combination significantly suppresses primary tumor growth, elicits systemic antitumor immunity and durable immunological memory, completely preventing post-surgical tumor recurrence.
• This strategy demonstrates strong synergy with PD-L1 checkpoint blockade, offering a new avenue to overcome immunotherapy resistance in hepatocellular carcinoma.

## Graphical abstract

**Figure:**
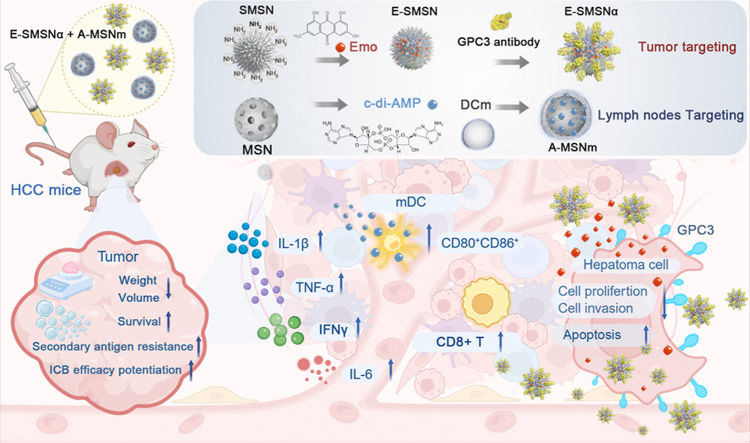

## 1 Introduction

Hepatocellular carcinoma (HCC) presents a critical global health challenge with progressively increasing incidence and mortality [[1], [2], [3], [4]]. Although conventional treatments, including surgical resection, liver transplantation, and chemoembolization, have been widely applied, their efficacy remains limited [5,6]. Recently, immune checkpoint blockade (ICB), particularly PD-1/PD-L1 inhibitors, has revolutionized HCC management and induced durable responses in a subset of patients [[7], [8], [9]]. However, objective response rates remain limited to 15–20%, with complete responses observed in fewer than 5% of cases [[10], [11], [12]]. One major factor contributing to this constrained efficacy is the inherently low immunogenicity of HCC tumors, which is associated with poor prognosis [13]. Additionally, impaired dendritic cell (DC) function fails to adequately prime naïve T cells, leading to compromised T-cell-mediated antitumor immunity in HCC patients [[14], [15], [16]]. Therefore, it is highly desired to develop novel immunotherapeutic strategies to simultaneously enhance tumor-associated antigen (TAA) release and re-shape the dysfunctional DC status.

Chemotherapy has traditionally been used to improve the immunogenicity of tumors. However, its application in HCC meets the challenges of low response rates and serious adverse effects, highlighting the urgent need for superior TAA release inducers [17]. Emodin, a natural compound from *Rheum palmatum,* exhibits multifaceted tumor-suppressive effects across multiple malignancies [18,19], including the induction of apoptosis and inhibition of proliferation. These properties establish it as a promising tumoricidal module for generating TAA to enable immune recognition. In parallel, DC activation can be achieved through multiple strategies, such as pulsing with tumor lysates, engaging pattern recognition receptors, or activating the cGAS-STING pathway, with the latter being particularly critical for DC maturation [[20], [21], [22]]. As a well-established STING agonist, c-di-AMP has been widely demonstrated to trigger this pathway [23,24]. Therefore, we reasoned that a combination strategy specifically delivering emodin to tumor cells and c-di-AMP to DCs may effectively overcome the therapeutic barrier in HCC.

**Fig. 1:**
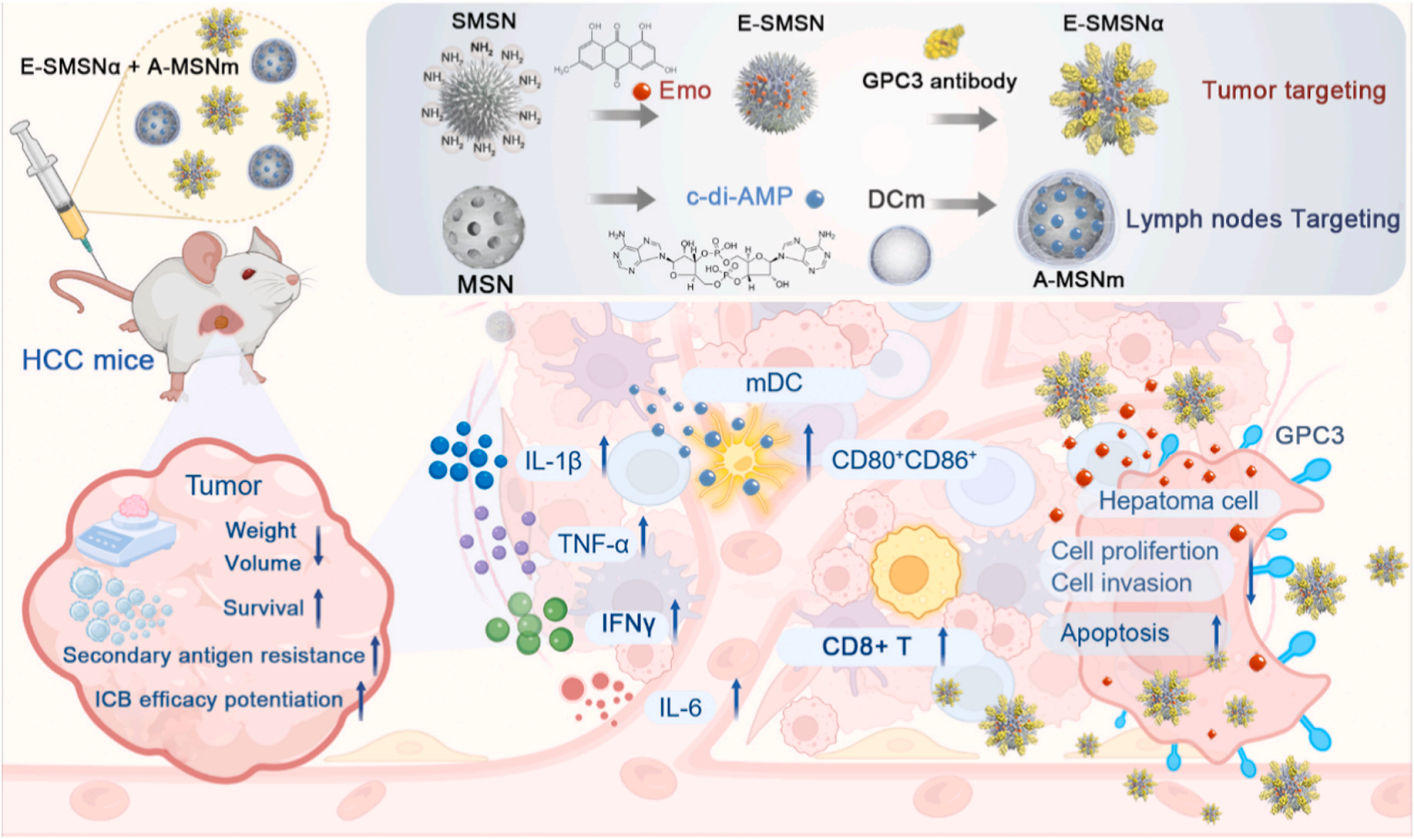
Schematic diagram illustrating the synergistic therapeutic mechanism of the nanoparticle dyad in HCC model. E-SMSNα leverages GPC3 expression on HCC cells to achieve tumor-specific targeting and deliver emodin, which directly inhibits cell proliferation and invasion while inducing apoptosis to release TAA. In parallel, A-MSNm exploits the inherent homing properties of matured BMDC membranes to tumor-draining lymph nodes, where it reprograms the dysfunctional DCs. This combination strategy elicits a robust antitumor immune response, resulting in significant tumor growth inhibition and long-lasting immunity against tumor rechallenge. Moreover, it potentiates the efficacy of ICB.

Here, we developed a nanoparticle dyad, for combinatorial HCC therapy. It comprises an anti-GPC3-antibody wrapped, emodin-loaded spiky MSN (E-SMSNα) for the highly efficient elimination of HCC cells, and a matured DC-membrane-camouflaged, c-di-AMP-loaded MSN (A-MSNm) for DC activation. The E-SMSNα capitalizes on GPC3 in HCC cells to enhance tumor-specific targeting and emodin delivery, thereby aiming to maximize direct tumor cell elimination and the consequent release of tumor antigens. In parallel, the A-MSNm exploits the inherent homing properties of matured bone marrow-derived dendritic cells (BMDCs) to lymph nodes, where it reprograms the tolerogenic DC status, thereby bridging innate and adaptive immunity. This combinatory regimen induces a potent immune response, thereby dramatically inhibiting tumor growth and conferring durable protection against secondary tumor challenge. Furthermore, it demonstrates strong synergy with PD-1 checkpoint blockade, positioning it as a promising multimodal strategy for HCC treatment (Fig. 1).

## 2 Results

### 2.1 E-SMSNα and A-MSNm preparation and characterization

**Fig. 2:**
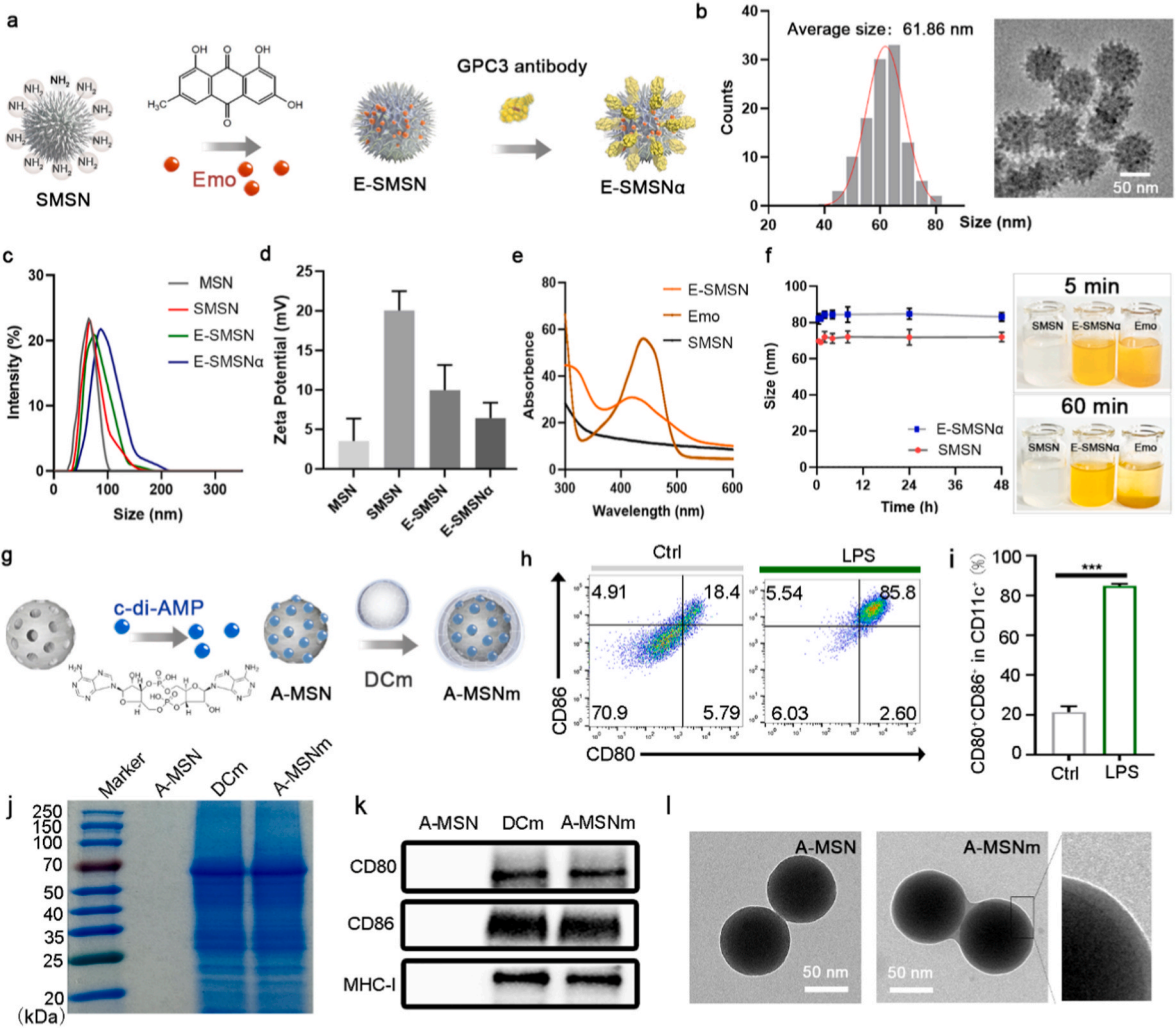
Preparation and characterization of E-SMSNα and A-MSNm. (a) Schematic illustration to show the preparation of E-SMSNα. (b) Representative images of E-SMSNα by TEM. Scale bar = 50 nm (c). Hydrodynamic size distribution of unloaded MSN, SMSN, E-SMSN, and E-SMSNα determined by DLS. (d) Zeta potential of MSN, SMSN, E-SMSN, and E-SMSNα measured by DLS. (e) The UV-vis absorption spectra of unloaded SMSN, free Emodin and E-SMSN. (f) Left panel: Size stability of SMSN and E-SMSNα stored at room temperature over 48 h. Right panel: Representative photographs of SMSN, E-SMSNα, and free emodin dispersions after 5 min and 60 min of storage. (g) Schematic illustration to show the preparation of A-MSNm. (h) Flow cytometric analysis of CD80^+^CD86^+^ expression on BMDCs stimulated with LPS compared to unstimulated control. (i) Quantification of CD80^+^CD86^+^ cells among CD11c^+^ BMDCs in each group. (j) SDS-PAGE analysis of protein profiles from bare A-MSN, native dendritic cell membrane (DCm), and membrane-coated A-MSN (A-MSNm). (k) Western blot analysis of CD80, CD86, and MHC-I expression on A-MSN, DCm, and A-MSNm. (l) Representative images of A-MSN and A-MSNm by TEM. Data are represented as mean ± SD (n = 3). Statistical significance was calculated via two-tailed Student's *t*-test, ***p < 0.001.

To augment the internalization of emodin, we developed E-SMSNα through a multi-step preparation process. First, bare mesoporous silica nanoparticles (MSN) were synthesized via a heterogeneous oil–water biphasic reaction. [[25], [26], [27]]. The MSN surface was then aminated by treatment with 3-aminopropyltriethoxysilane (APTES), yielding spiky mesoporous silica nanoparticles (SMSN) [[28], [29], [30]]. Emodin was subsequently loaded into the SMSN to form E-SMSN, followed by surface functionalization with DSPE-PEG-conjugated anti-GPC3 antibody, resulting in the final construct E-SMSNα (Fig. 2a).

E-SMSNα exhibited a spherical morphology with a uniform particle size of 61.86 nm (Fig. 2b). The stepwise assembly of the nanoparticle was monitored by dynamic light scattering (DLS) and zeta-potential measurements. Hydrodynamic diameters increased progressively from bare MSN (60.99 nm) to SMSN (69.81 nm), E-SMSN (76.8 nm), and E-SMSNα (82.56 nm) (Fig. 2c). The zeta potential of bare MSN was +3.5 mV. Due to amination modification, SMSN showed a markedly higher positive charge (+20.06 mV). Loading with emodin reduced the zeta potential of E-SMSN to +9.98 mV, and subsequent antibody decoration yielded a final value of +6.43 mV for E-SMSNα (Fig. 2d). Successful immobilization of antibodies was confirmed using a fluorophore-labeled antibody, which produced intense fluorescence upon 405 nm laser irradiation (Fig. S1). Critically, the successful loading of emodin into the nanoparticles was verified by its characteristic UV absorption peak at 440 nm (Fig. S2 and 2e). Based on this signature, the maximum drug loading capacity in E-SMSN was quantified to be 27.03 ± 1.25% (Fig. S3a). The encapsulation efficiency was 42.37 ± 6.25% at this optimal ratio. The emodin release profile showed pH-dependent behavior, with significantly enhanced release at the tumor-relevant acidic pH 5.5 (53.03% ± 2.6%) compared to pH 7.4 and pH 6.8 (Fig. S3b). For A-MSNm, the c-di-AMP loading capacity increased with higher weight ratios and plateaued at 1:1, with an encapsulation efficiency of 43.16 ± 2.17% (Fig. S3c). The c-di-AMP release profile also demonstrated faster release at pH 5.5, reaching approximately 53.54% (Fig. S3d). Furthermore, in contrast to free emodin, the SMSN and E-SMSNα formulation demonstrated excellent stability when stored at room temperature for 48 h (Fig. 2f).

To enable DC activation, A-MSNm was synthesized by loading MSN with cyclic di-AMP (c-di-AMP), followed by the camouflage with matured bone marrow-derived dendritic cells-membrane (DCm) (Fig. 2g). To verify the mature phenotype of BMDCs before membrane extraction, we analyzed LPS-stimulated BMDCs by flow cytometry. The results confirmed that over 80% of BMDCs were double-positive for CD80 and CD86, indicating efficient maturation (Fig. 2h and i). We then extracted the cell membrane from these mature BMDCs and performed SDS-PAGE analysis. The protein band pattern of the membrane-coated A-MSN (A-MSNm) was highly consistent with that of native DC membranes, indicating successful retention of the overall membrane protein profile (Fig. 2j). Furthermore, we performed Western blot analysis specifically for CD80 and CD86. The results demonstrated that both CD80 and CD86 were highly expressed on the mature DC membrane and A-MSNm, while bare A-MSN showed no detectable bands (Fig. 2k). These data collectively confirm that BMDCs were fully mature before membrane extraction and that key costimulatory molecules were effectively retained on the A-MSNm.

Having confirmed the successful retention of key membrane proteins, we next examined the structural and physicochemical properties of A-MSNm. TEM confirmed a well-defined core–shell morphology, indicative of successful membrane coating (Fig. 2l). Energy-dispersive X-ray spectroscopy elemental mapping of A-MSNm demonstrated a distinct spatial distribution of key constituents with Si and O originating from the MSN core while P and S, indicative of the membrane, primarily localized at the periphery. N, derived from both c-di-AMP and membrane proteins, was uniformly distributed throughout the nanoparticle, providing direct evidence for the successful integration of all components (Fig. S4). Dynamic light scattering (DLS) analysis indicated that the hydrodynamic sizes of MSN, A-MSN, and A-MSNm were 61.57 ± 2.56 nm, 75.07 ± 2.76 nm, and 80.51 ± 1.89 nm, respectively (Fig. S5a). Zeta potential measurements showed that the surface charge of MSN was +3.5 ± 2.86 mV, which shifted to −5.26 ± 1.07 mV after loading with c-di-AMP to form A-MSN, and further decreased to −26.17 ± 1.05 mV upon matured BMDC membrane coating to obtain A-MSNm (Fig. S5b). Upon storage in serum for 48 h, A-MSNm retained its original zeta potential and size, indicating superior stability (Fig. S5c–d).

### 2.2 Biocompatibility and targeting of the nanocarriers

We following evaluated the biocompatibility of the nanocarriers SMSNα and MSNm using 293T cells. Both carriers exhibited negligible effects on cell viability across a concentration range of 10 μg mL^−1^ to 150 μg mL^−1^ (Figs. S6 and S7). Moreover, treating 293T cells with 100 μg mL^−1^ SMSNα or 100 μg mL^−1^ MSNm resulted in stable viability within 48 h (Figs. S8 and S9). These data demonstrate that both SMSNα and MSNm possess good biocompatibility for subsequent applications.

We next investigated the targeted delivery potential of the GPC3 antibody-modified platform, SMSNα. Consistent with previous findings [31], we confirmed high expression of the GPC3 antigen in human HCC cell lines, including HepG2, Hep3B, and Huh7 (Fig. S10a). To evaluate targeting specificity, FITC-labeled emodin was loaded into SMSN particles with or without GPC3α modification. Fluorescence analysis in HepG2 cells revealed that the GPC3α-modified particles exhibited significantly greater cellular association at both 40 and 90 min compared to the unmodified control (Fig. S10b–c), demonstrating enhanced targeting to HCC cells *in vitro*. We further performed the same uptake assay in mouse Hepa1-6 cells. E-SMSNα exhibited significantly greater cellular association at both 40 and 90 min compared to non-targeted E-SMSN, and this enhanced uptake was abolished by competitive blocking with free anti-GPC3 antibody, confirming specific receptor-mediated internalization in Hepa1-6 cells (Fig. S11).

We further assessed the *in vivo* targeting capability of E-SMSNα using a mouse model bearing HepG2 tumor xenografts. After intravenous injection via the tail vein with E^FITC^-SMSNα, fluorescence imaging was conducted at both 6 h and 24 h post-injection to visualize the distribution and accumulation of the nanoparticles. The E^FITC^-SMSNα group demonstrated significantly higher fluorescence signals at the tumor site compared to the free E^FITC^ group, indicating successful accumulation of E-SMSNα at the tumor (Fig. S12a). Additionally, emo concentration in the E-SMSNα group decreased more slowly than in the free Emo group, suggesting that E-SMSNα has a longer circulation time in the bloodstream (Fig. S12b). To further validate the targeting capability of SMSNα, we performed additional verification in Hepa1-6 hepatocellular carcinoma-bearing mice. E-SMSNα exhibited significantly stronger and more sustained tumor fluorescence signals at both 6 h and 24 h post-injection compared to non-targeted E-SMSN, confirming that anti-GPC3 antibody modification enhances tumor accumulation in this syngeneic HCC model (Fig. S13). This prolonged circulation time is likely due to the protection of Emo by the SMSNα coating, which reduces its clearance by the reticuloendothelial system. The extended circulation time of E-SMSNα could enhance its therapeutic efficacy by allowing more time for accumulation at the tumor site.

Next, to evaluate the *in vivo* distribution and lymph node homing capacity of A-MSNm, we labeled A-MSN and A-MSNm with Cy7 and injected them subcutaneously into Hepa1-6 tumor-bearing mice. Ex vivo imaging of major organs, tumors, and tumor-draining lymph nodes (TDLNs) revealed that Cy7-MSNm showed significantly higher accumulation in TDLNs compared to Cy7-MSN, confirming that the matured DC membrane coating confers superior lymph node targeting (Fig. S15).

Having confirmed the respective functionalities of E-SMSNα in tumor targeting and A-MSNm in lymph node homing, we next evaluated their *in vivo* biocompatibility and potential toxicity. To examine the safety of the E-SMSNα and A-MSNm, the major organs of the mice were collected for histological analysis. Hematoxylin and eosin (H&E) staining of major organ sections revealed no significant morphological damage (Fig. S14). To further evaluate the systemic safety of the nanoparticle dyad, serum biochemical and hematological parameters were measured in Hepa1-6 tumor-bearing mice after treatment. Serum levels of alkaline phosphatase (ALP) and alanine aminotransferase (ALT), as well as complete blood count parameters including platelets (PLT), red blood cells (RBC), and white blood cells (WBC), showed no significant differences compared to the control group (Fig. S16). These results demonstrate the superior *in vitro* and *in vivo* targeting performance of the SMSNα platform for HCC.

### 2.3 E-SMSNα acts as a potent tumoricidal module

**Fig. 3:**
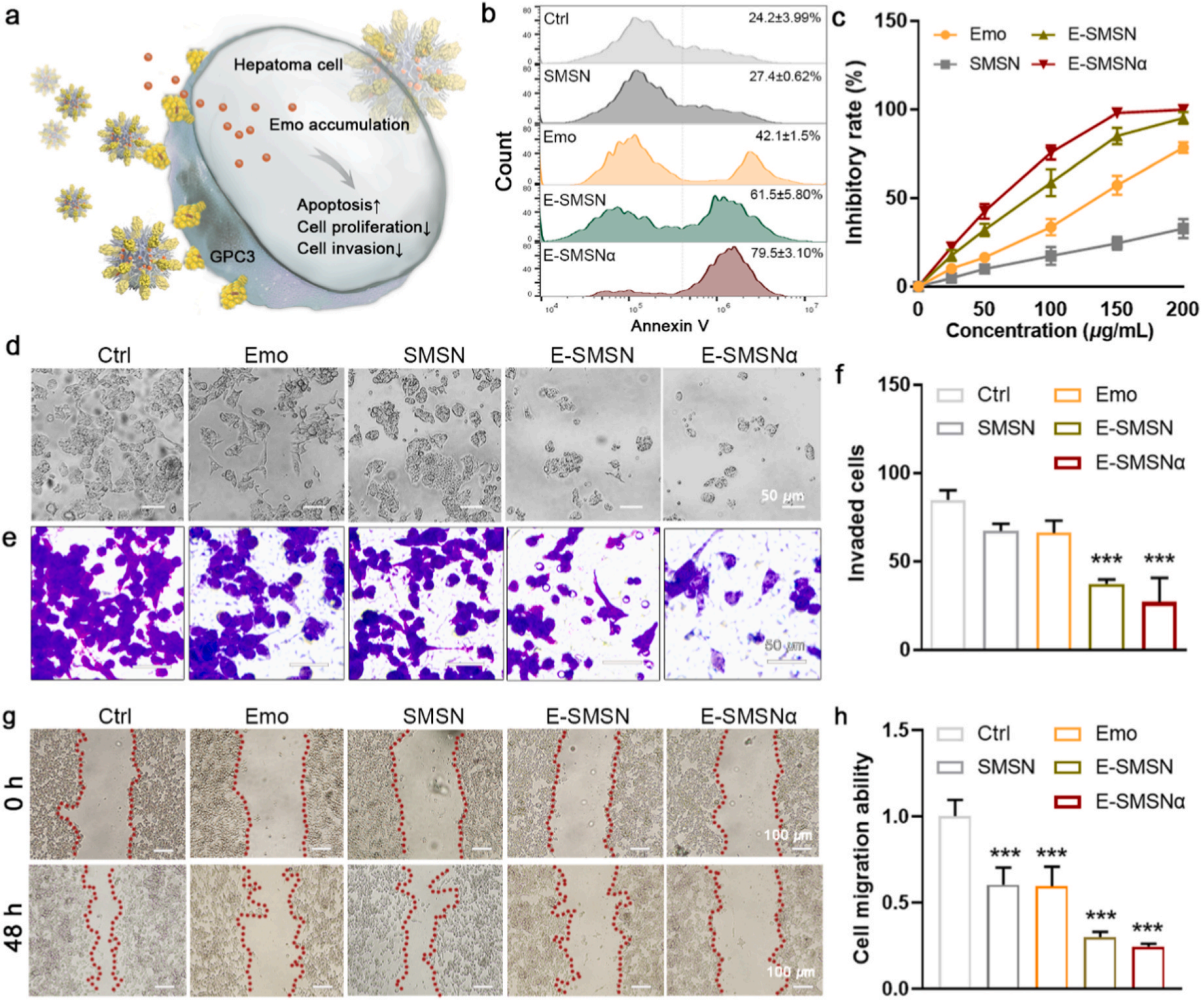
**E-SMSNα eliminates HCC cells.** (a) Schematic illustration of the proposed mechanisms by which E-SMSNα may affect HCC cells. (b) Flow cytometry analysis showing the percentage of annexin V^+^ cells. (c) Determination of the inhibitory rate of Emo, SMSN, E-SMSN and E-SMSNα in HepG2 cells by CCK8 assay. (d) Representative bright-field microscopy imaging of HepG2 cells treated with Emo, SMSN, E-SMSN, or E-SMSNα. (e) The effects of E-SMSNα on cell invasion. (f) Quantification of (e). (g) The effects of E-SMSNα on cell migration. The contour of cell migration was labeled by dashed lines. (h) Quantification of (g). Data are represented as mean ± SD (n = 3). One-way ANOVA with Tukey's post-hoc test, *p < 0.05, **p < 0.01, ***p < 0.001.

Having validated the targeting capability of E-SMSNα, we next assessed its efficacy as the tumoricidal module on HCC cells (Fig. 3a). Previous studies have indicated that emodin promotes apoptosis in HCC cells and inhibits their proliferation and migration, thereby exerting anticancer effects [32,33]. To evaluate the apoptotic-inducing effects of E-SMSNα on HCC cells, we used flow cytometry to measure the levels of apoptosis in HepG2 cells treated with SMSN, emodin, E-SMSN, and E-SMSNα. The results showed that the proportion of apoptotic HCC cells increased by 37.3% for E-SMSN and 55.3% for E-SMSNα compared to the control group (Fig. 3b–S17). We performed the same apoptosis assay in Hepa1-6 cells. E-SMSNα induced a marked increase in apoptotic cells, with the potency following the same order as observed in HepG2 (Fig. S18). To investigate the inhibitory effects of these nanoformulas, we conducted a CCK8 assay in HepG2 cells. The results revealed that the half-maximal inhibitory concentration (IC50) of SMSN was greater than 200 μg/mL. In contrast, Emo exhibited an IC50 of 100 to 150 μg/mL. Notably, both E-SMSN and E-SMSNα demonstrated significantly lower IC50 values, ranging from 50 to 100 μg/mL, with E-SMSNα showing a more potent inhibitory effect (Fig. 3c). This finding was further corroborated by bright-field microscopy imaging of the cells (Fig. 3d). Furthermore, since metastasis suppression is another key anticancer effect of Emodin, we evaluated the impact of our formulations on HCC cell invasion and migration. Transwell and wound healing assays showed that E-SMSNα most effectively inhibited the invasive and migratory capacities of HepG2 cells (Fig. 3e–h). To ensure consistency between the *in vitro* and *in vivo* models, we performed the same assays in Hepa1-6 cells. E-SMSNα most effectively inhibited the invasive and migratory capacities of Hepa1-6 cells, consistent with the results observed in HepG2 cells (Fig. S19). These results demonstrate the efficient elimination and containment of HCC cells by E-SMSNα.

To further investigate whether E-SMSNα can induce immunogenic cell death (ICD) in HCC, we examined key hallmarks of ICD in Hepa1-6 cells after treatment. The results showed that E-SMSNα treatment significantly increased cell surface exposure of calreticulin (CRT), reduced intracellular high-mobility group box 1 (HMGB1) levels, and elevated ATP release into the supernatant (Fig. S20). These findings collectively demonstrate that E-SMSNα induces ICD in HCC cells, which is essential for subsequent immune recognition.

### 2.4 Immune regulation of A-MSNm

To investigate whether A-MSNm can efficiently reprograms DCs, we first assessed its ability to activate the STING pathway in immatured BMDCs. It has been well-documented that c-di-AMP directly binds to STING, leading to the phosphorylation of IRF3 (p-IRF3) and driving the secretion of Type I interferons. We performed Western blot analysis to examine the levels of total STING, p-STING, total IRF3, and p-IRF3 in BMDCs treated with c-di-AMP or A-MSNm. Compared to the control group, A-MSNm treatment significantly enhanced p-STING and p-IRF3 expression, while free c-di-AMP also induced activation but to a lesser extent (Fig. S21). Similar results were found in p-IRF3 immunofluorescence analysis (Fig. S22), demonstrating successful activation of the STING signaling axis.

**Fig. 4:**
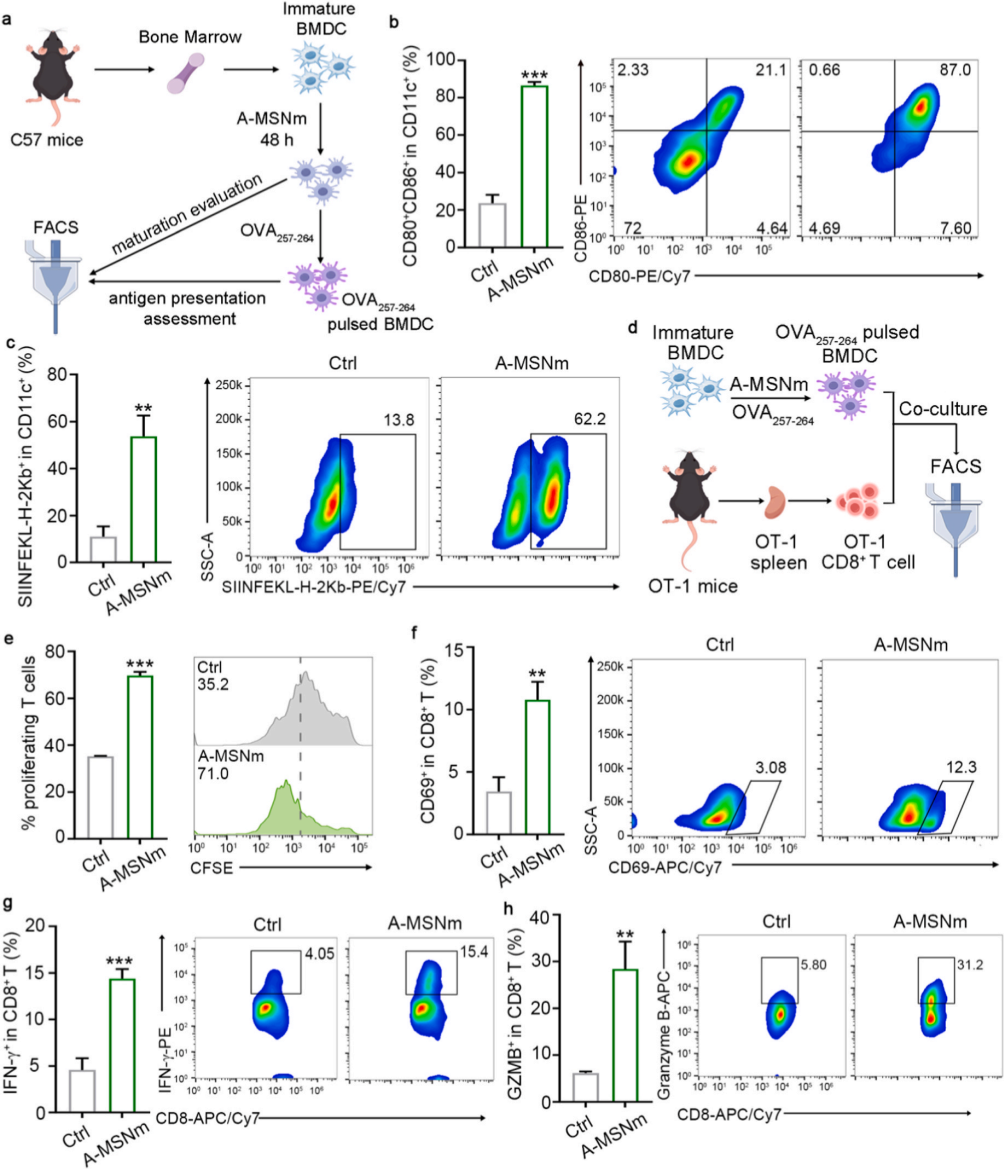
**Immune regulation of A-MSNm *in vitro*.** (a) Experimental design for assessing BMDC maturation and antigen presentation. (b,c) FACS analysis the proportions of CD80^+^CD86^+^ BMDCs (gated on CD11c^+^ cells) (b) and SIINFEKL-H-2Kb^+^ BMDCs (gated on CD11c^+^ cells) in different groups (c). (d) Schematic illustration of the BMDC-T cell co-culture system for T cell function analysis. (e) Measurement of CD8^+^ T cell proliferation by CFSE dilution analysis. (f-h) FACS analysis results of CD69^+^CD8^+^ T cells (f), IFN-γ^+^CD8^+^ T cells (g) and GZMB^+^CD8^+^ T cells (h) after different treatments. Data are shown as mean ± SD (n = 3). Complete gating strategies for flow cytometric analyses are provided in Fig. S24. Statistical significance was calculated via two-tailed Student's *t*-test (b-c, e-h), **p < 0.01, ***p < 0.001.

Since STING signaling is a key trigger for DC maturation, we next asked whether this activation functionally promotes DC maturation (Fig. 4a). We focused on the A-MSNm group, as it represents our most therapeutically relevant formulation. FACS analysis revealed that A-MSNm treatment significantly increased the proportion of mature DC (CD80^+^CD86^+^), resulting in approximately a 3.7-fold higher frequency compared to the control group (Fig. 4b). To further confirm that the observed DC maturation was driven by STING signaling rather than residual LPS or membrane-derived inflammatory signals, we treated BMDCs with MSNm, A-MSNm, or A-MSNm combined with the STING inhibitor C176 (A-MSNm + C176), and measured IFN-β production and expression of downstream interferon-stimulated genes (ISGs) including Irf-7, Ifit1, Isg15, Oasl1, and Ccl5. A-MSNm significantly induced IFN-β and all tested ISGs compared to MSNm, confirming that c-di-AMP loading drives STING pathway activation. The addition of C176 almost completely abolished these responses, indicating that the STING pathway is the primary mediator of the observed innate activation in BMDCs (Fig. S23).

To assess MHC-I peptide presentation capacity, BMDCs pre-treated with A-MSNm were pulsed with the OVA_257_–_264_ peptide (Fig. 4a). The proportion of SIINFEKL–H-2Kb^+^ BMDCs in the A-MSNm group was approximately 4.9-fold higher than that in the control group, indicating a substantial enhancement in MHC-I-mediated antigen presentation (Fig. 4c). For functional validation, these antigen-pulsed DCs were co-cultured with CFSE-labeled OT-I CD8^+^ T cells at a DC: T cell ratio of 1:10 for 72 h (Fig. 4d). A-MSNm-matured DCs induced robust T cell proliferation, with the proportion of divided T cells being about 2-fold higher than that in the control group, as quantified by CFSE dilution (Fig. 4e). In addition, compared to the control group, a 3.1-fold elevation in CD69^+^CD8^+^ T cell population was observed in A-MSNm group, indicating enhanced early T cell activation (Fig. 4f). Intracellular cytokine staining showed markedly increased production of IFN-γ and granzyme B (GZMB) in CD8^+^ T cells in A-MSNm group, confirming enhanced effector cytotoxic function (Fig. 4g and h). Together, these findings indicate that A-MSNm potentiates STING-dependent signaling in DCs, which in turn promotes their maturation and significantly amplifies antigen-specific CD8^+^ T cell priming, proliferation, and functional differentiation.

While the above peptide-pulsing experiments confirmed that A-MSNm enhances MHC-I peptide presentation and subsequent T cell activation, they bypass the natural processes of antigen uptake, processing, and cross-presentation from tumor cells. To directly assess whether A-MSNm promotes the cross-presentation of tumor-derived antigens in a more physiological setting, BMDCs pre-treated with or without A-MSNm were co-cultured with E-MSNα-treated Hepa1-6-OVA tumor cells (dying tumor cells) for 24 h (Fig. S25a). We first measured the proportion of SIINFEKL–H-2Kb^+^ BMDCs to evaluate antigen uptake, processing, and cross-presentation. After removing un-engulfed tumor cells, the BMDCs were collected and co-cultured with OT-I CD8^+^ T cells, and T cell activation was assessed by CD69 expression (Fig. S25a). Compared to the control group, A-MSNm-treated BMDCs showed significantly higher SIINFEKL–H-2Kb^+^ presentation and induced stronger CD8^+^ T cell activation (Fig. S25b–c). These results provide direct evidence that A-MSNm enhances the cross-presentation of tumor-derived antigens released by dying tumor cells, rather than only facilitating peptide loading.

To further substantiate the claim that A-MSNm reshapes tolerogenic DCs *in vivo*, we performed additional experiments in Hepa1-6 tumor-bearing mice treated with PBS or A-MSNm. We analyzed DCs from both tumor-draining lymph nodes (TDLNs) and tumor tissues (Figs. S26–28). In the TDLNs, we evaluated DC activation by measuring CD11c^+^CD80^+^CD86^+^ cells as a general maturation marker (Fig. S26a), CD11c^+^MHC-II^+^CD40^+^ cells to assess antigen presentation and co-stimulatory capacity (Fig. S26b), and CD11c^+^CCR7^+^ cells to indicate DC homing to lymph nodes for T cell priming (Fig. S26c). In the tumor tissue, we examined CD11c^+^MHC-II^+^CD40^+^ DCs to evaluate the activation status of tumor-infiltrating DCs (Fig. S26d), and CD11c^+^IL-12^+^ cells to assess their capacity to produce IL-12, a key cytokine that drives Th1 differentiation and cytotoxic T cell activation (Fig. S26e). In all these analyses, A-MSNm treatment resulted in significantly higher frequencies compared to the control group (Fig. S26). These *in vivo* findings collectively provide direct evidence that A-MSNm reshapes tolerogenic DCs by converting tolerogenic DCs into fully activated, immune-promoting cells.

### 2.5 Transcriptomics analysis reveals tumor microenvironment reprogramming by the nanoparticle dyad

**Fig. 5:**
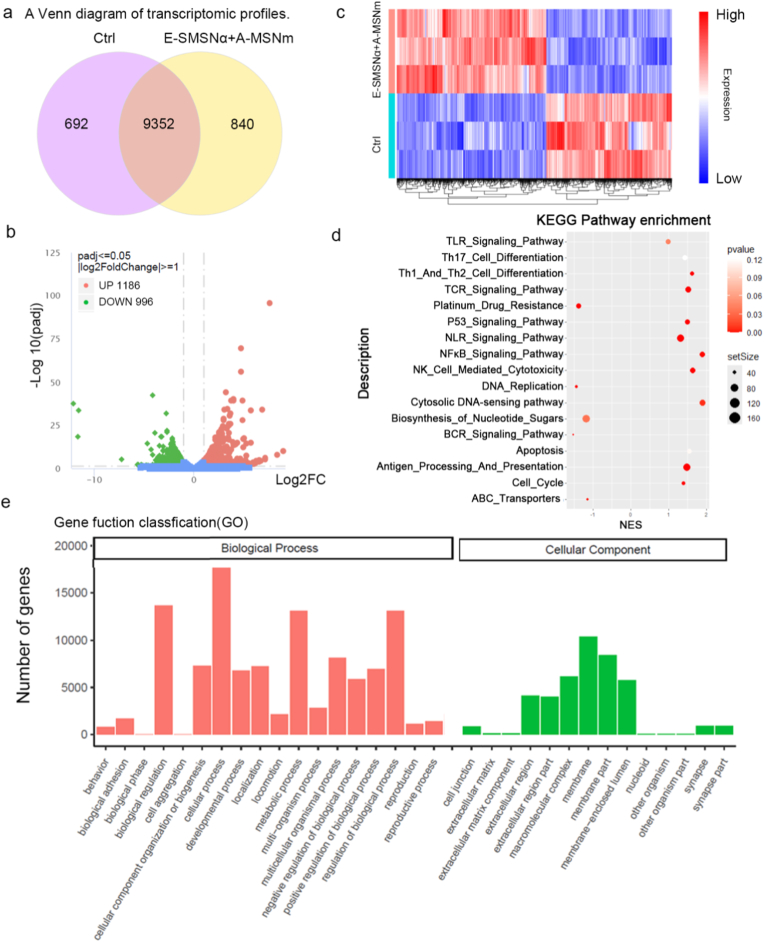
**Comprehensive transcriptome analysis of Hepa1-6 tumor-bearing mice treated with E-SMSNα+A-MSNm.** (a) Venn diagram showing the overlap of transcriptional profiles between the untreated and E-SMSNα+A-MSNm treated groups. (b) Volcano plot illustrating the differentially expressed genes (DEGs) with significant changes (padj <0.05 and |log2FoldChange| > 1). Green dots denote downregulated genes, while red dots represent upregulated genes. (c) Heatmap representing the differential gene expression between the Ctrl and E-SMSNα+A-MSNm groups. The color gradient from blue to red signifies low to high expression levels, respectively. (d) KEGG pathway enrichment analysis for the DEGs, displaying the pathways with significant enrichment. The plot shows the NES on the x-axis and the pathways on the y-axis, with dot size indicating geneSize and color representing p.adjust. (e) Gene Ontology (GO) enrichment analysis showing the classification of DEGs into biological processes and cellular components.

To further elucidate the therapeutic mechanism of the nanoparticle dyad at the genetic level, we performed transcriptome profiling of tumor tissues from Hepa1-6 tumor-bearing mice. Differential expression analysis was initially illustrated through a Venn diagram (Fig. 5a) and a volcano plot (Fig. 5b). A total of 2182 differentially expressed genes (DEGs) were identified, comprising 1186 upregulated genes and 996 downregulated genes. The heatmap analysis further revealed significant differences in mRNA expression between the control group and the nanoparticle dyad treated group (Fig. 5c). To interpret these changes functionally, we conducted Kyoto Encyclopedia of Genes and Genomes (KEGG) and Gene Ontology (GO) analyses. KEGG analysis indicated that in the E-SMSNα+A-MSNm treated group, there was significant enrichment in pathways related to immune response and cell differentiation, including the Cytosolic DNA-sensing pathway, NF-κB signaling pathway, NK cell-mediated cytotoxicity, Th1/Th2 cell differentiation, and BCR/TCR signaling pathway (Fig. 5d). Conversely, pathways associated with tumor cell survival and proliferation were downregulated. GO analysis further supported these findings, showing significant enrichment in biological processes such as immune response, cell proliferation, and cell cycle regulation, along with cellular components including ribosomes and extracellular regions (Fig. 5e). Together, these transcriptomic changes suggest that E-SMSNα+A-MSNm effectively reprograms the tumor immune microenvironment toward an anti-tumor phenotype, corroborating its role in enhancing therapeutic efficacy.

### 2.6 The nanoparticle dyad inhibits tumor progression in HCC

**Fig. 6:**
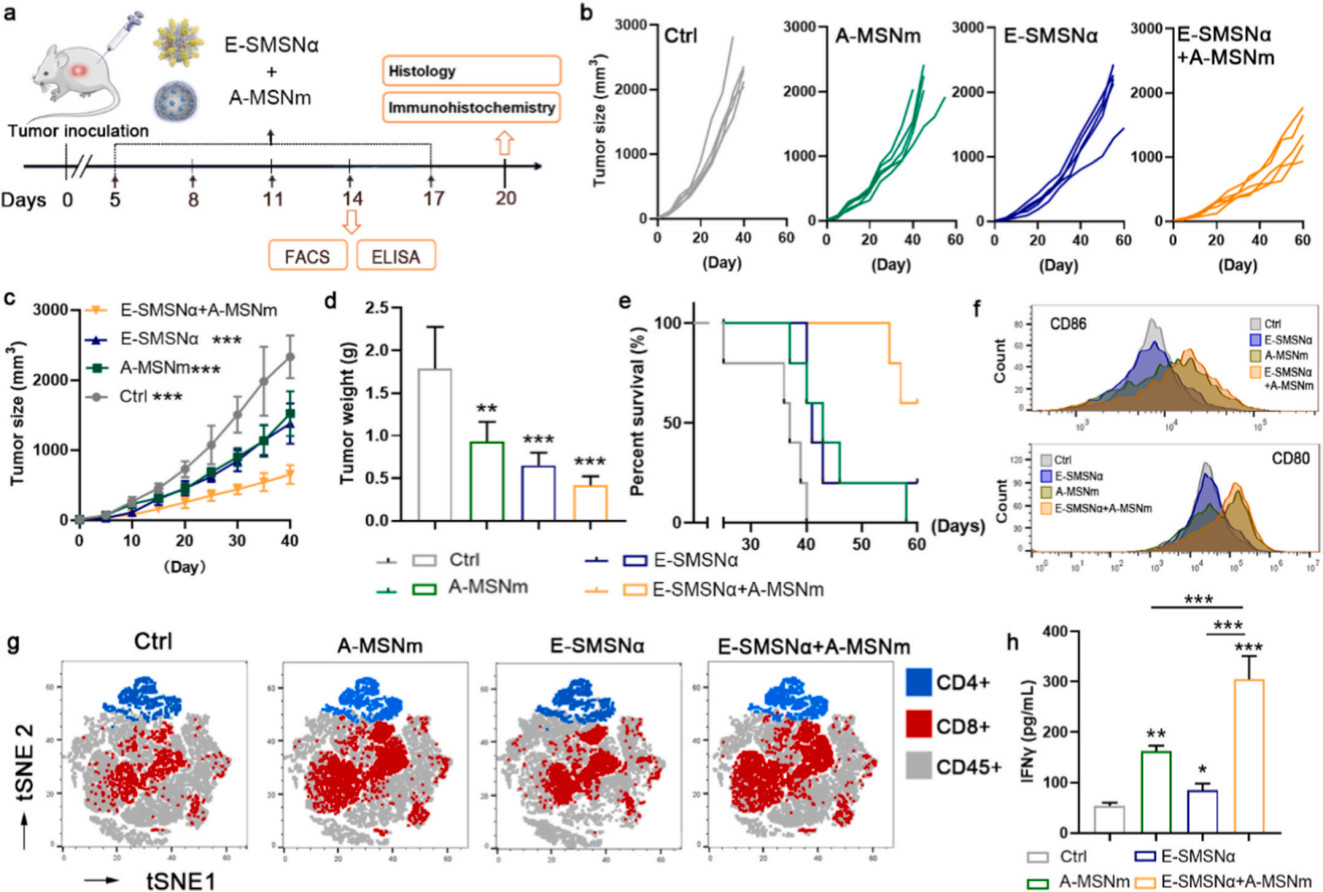
**The nanoparticle dyad inhibits tumor progression in Hepa1-6 hepatocellular carcinoma-bearing mice.** (a) Schematic representation of experimental design. (b) Individual tumor growth kinetics. (n = 5) (c) Average tumor growth curves. (d) Average tumor weight. (n = 5) (e) Animal survival curves. (n = 5) (f) Representative flow cytometric images of CD86^+^ and CD80^+^ cells in DCs. (g) Representative TSNE of CD8^+^ T cells from different treatment groups infiltrating into the tumor microenvironment. (h) Levels of serum IFNγ in tumor-bearing mice. (n = 3) Data are represented as mean ± SD. Two-way ANOVA with Tukey's post-hoc test for (c, d, h), Log-rank (Mantel–Cox) test for (e), *p < 0.05, **p < 0.01, ***p < 0.001.

In a further exploration of the therapeutic potential of the nanoparticle dyad, we conducted *in vivo* studies using Hepa1-6 hepatocellular carcinoma-bearing mice (Fig. 6a). Our findings unequivocally demonstrated that treatment with E-SMSNα and A-MSNm led to a significant reduction in tumor growth, as evidenced by decreased tumor volume and weight. On day 35 post-tumor implantation, the tumor volume in the treatment groups decreased from 1984.06 ± 490.08 mm^3^ to 1130.68 ± 229.31 mm^3^ (A-MSNm), 1138.72 ± 212.21 mm^3^ (E-SMSNα), and 544.82 ± 129.18 mm^3^ (E-SMSNα+A-MSNm), while the tumor weight reduced from 1.78 ± 0.49 g to 0.93 ± 0.22 g (A-MSNm), 0.65 ± 0.15 g (E-SMSNα), and 0.42 ± 0.11 g (E-SMSNα +A-MSNm) (Fig. 6b–d). After treatment with E-SMSNα+A-MSNm, the survival period of the mice was significantly prolonged, with the median survival time extending from 37 days to over 60 days (Fig. 6e).

Additionally, E-SMSNα and A-MSNm administration resulted in the activation of immune responses, characterized by an increased infiltration of immune cells such as DCs and T cells into the tumor microenvironment. On day 20, FACS indicated that the proportion of CD86^+^ cells among immune cells was 6.42 ± 0.62% in the Ctrl group. Following E-SMSNα administration, this proportion increased to 9.48 ± 0.41%. In the A-MSNm and A-MSNm + E-SMSNα groups, the proportion further increased to 31.97 ± 0.65% and 38.77 ± 0.55% (Fig. 6f–S29). Immunofluorescence staining of CD86 on mouse tumor tissue sections further confirmed this result (Fig. S30). Similarly, the proportion of CD80^+^ cells was 10.51 ± 1.14% in the Ctrl group, rising to 14.03 ± 0.70% after E-SMSNα administration. In the A-MSNm and A-MSNm + E-SMSNα groups, the proportion increased to 35.97 ± 0.67% and 53.17 ± 1.03% (Fig. 6f–S29). The proportion of CD8^+^ T cells among immune cells was 7.59 ± 2.71% in Ctrl group. Following E-SMSNα administration, this proportion increased to 12.19 ± 2.35%. In the A-MSNm and A-MSNm + E-SMSNα groups, the proportion more than doubled to 17.85 ± 4.7% and 26.23 ± 1.28% (Fig. 6g–S31). This immune activation was further supported by the secretion of pro-inflammatory cytokines. Specifically, the combination of E-SMSNα and A-MSNm markedly elevated the serum level of IFNγ to 305.02 ± 45.87 pg/mL, representing an increase of more than 8-fold over the control group (54.78 ± 5.89 pg/mL) (Fig. 6h). Similarly, compared to other treatment groups, stimulation with E-SMSNα+A-MSNm resulted in the greatest increase in the levels of TNF-α, IL-6, and IL-1β in the mouse serum (Fig. S32). Collectively, E-SMSNα+A-MSNm demonstrated the most effective tumor suppression and immune activation.

**Fig. 7:**
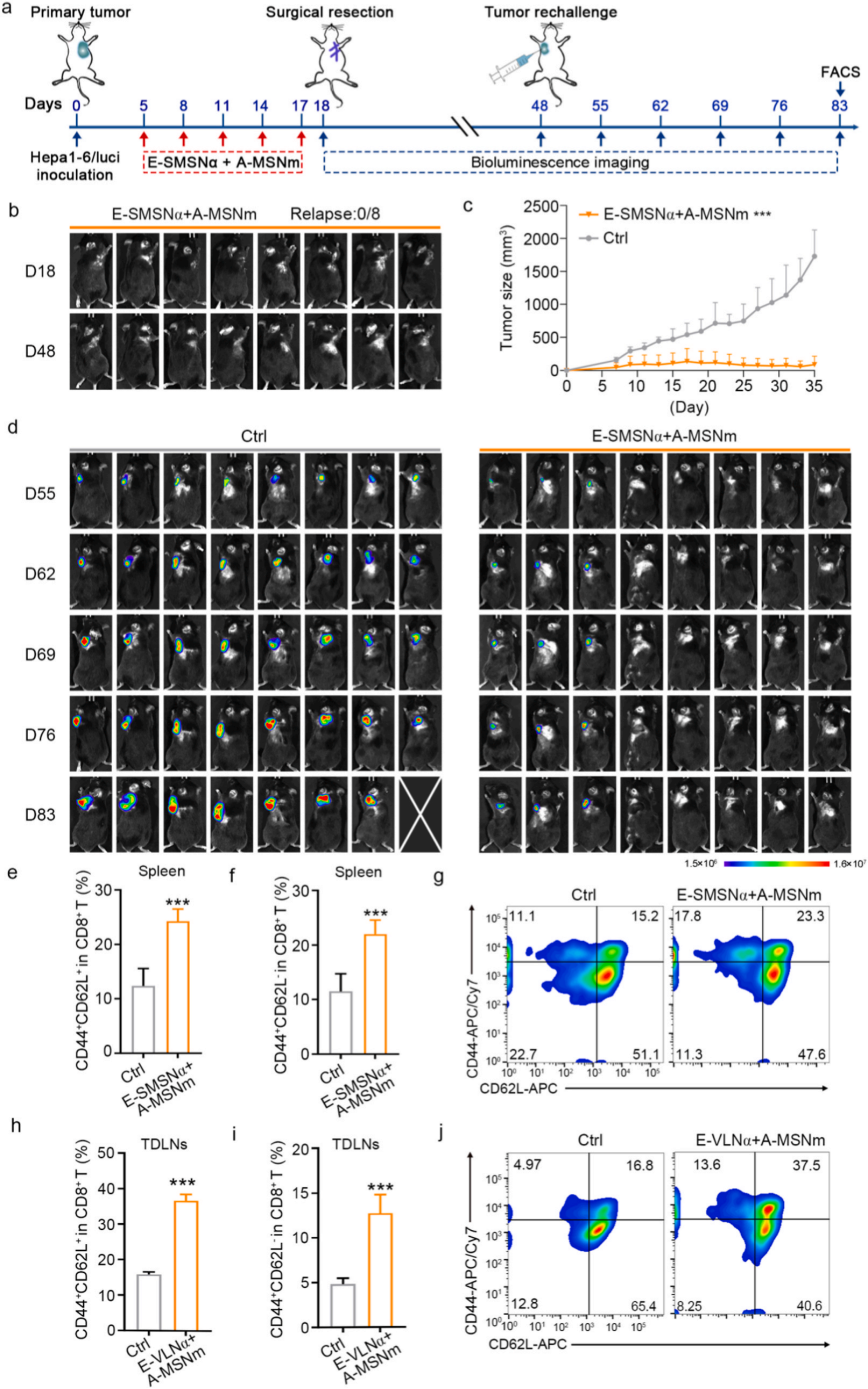
**E-SMSNα+A-MSNm enhances immunological memory and prevents tumor recurrence in Hepa1-6 tumor-bearing mice.** (a) Schematic illustration for tumors re-challenge studies. (b) *In vivo* monitoring of tumor elimination on day 0 and day 30 post primary tumor removal surgery (n = 8). (c) Average tumor growth curves (n = 8). Two-way ANOVA with Tukey's post-hoc test. (d) Bioluminescent tracking of Hepa1-6/luci re-challenge. (e-j) FACS analysis results of T_CM_ cells (CD44^+^CD62L^+^, gated on CD45^+^CD3^+^CD8^+^ cells) and T_EM_ cells (CD44^+^CD62L^−^, gated on CD45^+^CD3^+^CD8^+^ cells) in (e,f) spleens and (h,i) TDLNs. Data were shown as the mean ± SD (n = 5). (g) Representative flow cytometric images for (e) and (f). (j) Representative flow cytometric images for (h) and (i). Complete gating strategies for flow cytometric analyses are provided in Fig. S33. Statistical significance was calculated via two-tailed Student's *t*-test, **p < 0.01, ***p < 0.001.

To assess the establishment of antigen-specific immunological memory, we surgically removed the primary Hepa1-6 tumors on day 18 (Fig. 7a). Remarkably, tumor recurrence was effectively suppressed in all eight mice that had received the combined E-SMSNα+A-MSNm therapy (Fig. 7b). To stringently test the durability of this protective effect, we administered a secondary tumor challenge on day 48 using age-matched naïve mice as controls. While control mice exhibited 100% tumor progression by day 83, majority (5 out of 8) of the treated mice developed undetectable tumors. Even in these few cases, tumor growth was markedly restrained, showing a reduction in bioluminescent signal intensity and a significant delay in progression kinetics compared to controls (Fig. 7c and d). Further mechanistic analysis of the immune cells provided a mechanistic basis for these observations. We detected a substantial expansion of memory T cells (Tcm, CD45^+^CD3^+^CD8^+^CD44^+^CD62L^+^, Tem, CD45^+^CD3^+^CD8^+^CD44^+^CD62L^−^) in both spleens (Fig. 7e–g) and tumor-draining lymph nodes (TDLNs) (Fig. 7h–j) of the E-SMSNα+A-MSNm-treated group. Collectively, these findings demonstrate that the E-SMSNα+A-MSNm combination therapy not only achieves primary tumor clearance but also effectively primes a systemic and durable immunoprotective memory to combat secondary antigen encounters.

### 2.7 The nanoparticle dyad enables ICB therapy in HCC

**Fig. 8:**
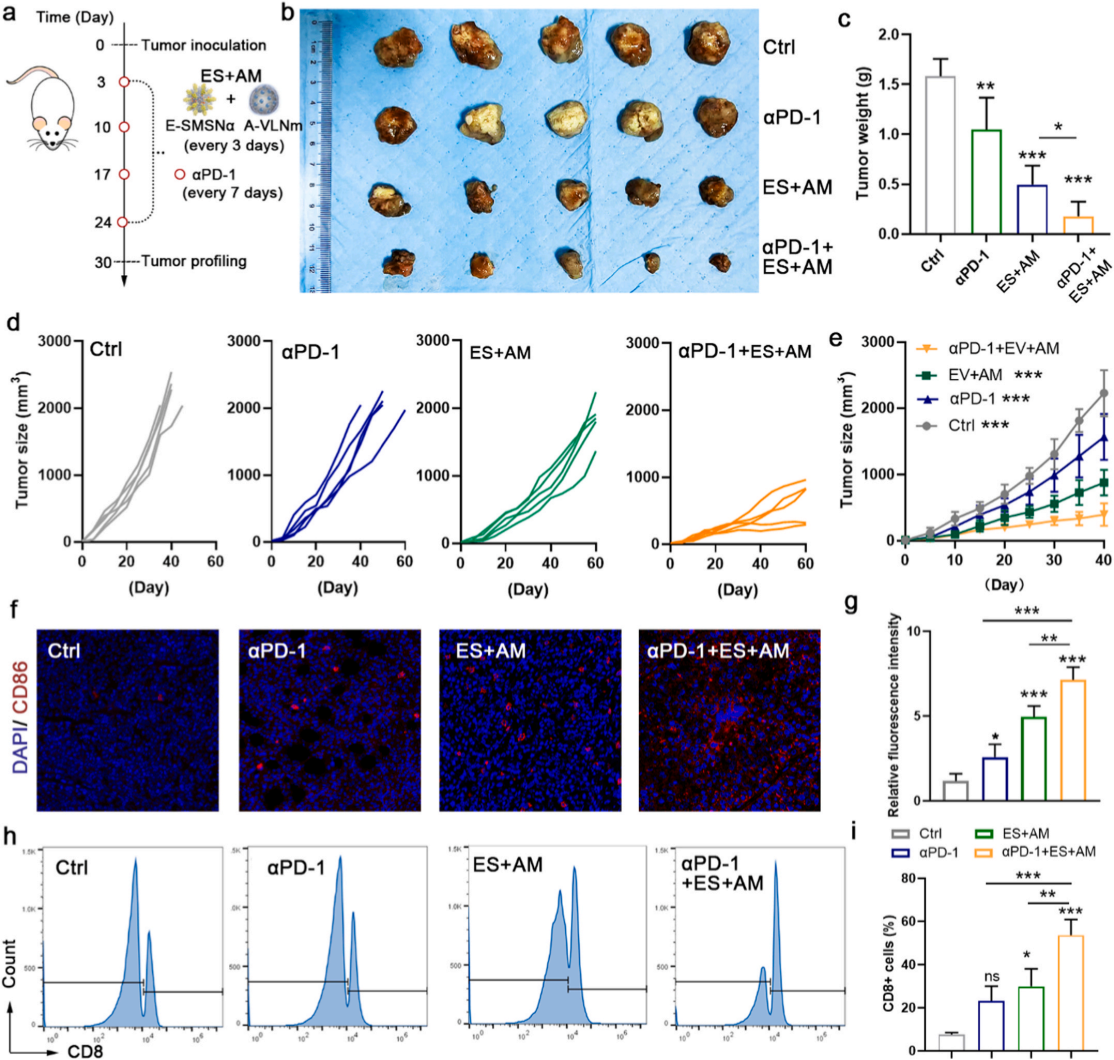
**αPD-1 synergizes with nanoparticle dyad to augment tumor suppression and immune activation in HCC.** (a) Schematic representation of experimental design for ES + AM and αPD-1 treatment in Hepa1-6 hepatocellular carcinoma-bearing mice. (b) (c) Average tumor weight. (n = 5) (d) Individual tumor growth kinetics. (n = 5) (e) Average tumor growth curves. (n = 5) (f) Representative fluorescence microscopy images showing DAPI (blue) and CD86 (red) staining in tumor sections for different treatment groups. (g) Quantification of CD86 fluorescence intensity. (n = 3) (h) Representative flow cytometric images of CD8^+^T (gated on CD45^+^ cells) in different groups. (i) Quantification of the percentages of CD45^+^CD8^+^ cells across groups. (n = 3) Data are represented as mean ± SD. Two-way ANOVA with Tukey's post-hoc test, ns: no significance, *p < 0.05, **p < 0.01, ***p < 0.001.

In follow-up experiments, we aim to further explore the potential of the nanoparticle dyad in combination with immune checkpoint inhibitors for the treatment of HCC. We further explored the potential of combining E-SMSNα+A-MSNm (ES + AM) and αPD-1. The dosing and treatment schedule for the mice is depicted in Fig. 8a. Tumor inoculation was performed at day 0, followed by treatments with ES + AM and αPD-1 every 3 days and every 7 days, until day 30 when tumor profiling was conducted. On day 30 post-tumor inoculation, the tumor weight in the αPD-1+ES + AM group was significantly lower compared to the control and other treatment groups (Fig. 8b and c). The tumor volumes at day 35 were 1812.06 ± 173.87 mm^3^ for Ctrl, 1277.05 ± 321.46 mm^3^ for αPD-1, 726.99 ± 189.26 mm^3^ for ES + AM, and 334.53 ± 100.87 mm^3^ for αPD-1+ES + AM (Fig. 8d and e). Compared to the control group, the tumor volume in the αPD-1+ES + AM treatment group was reduced by approximately 81.53%. These results indicate that the combination treatment was the most effective in reducing tumor volume. The combined administration of ES + AM and αPD-1 also led to the activation of immune responses, characterized by an increased infiltration of immune cells such as mature DCs and CD8^+^ T cells into the tumor microenvironment. Immunofluorescence staining of tumor tissues revealed that the αPD-1+ES+AM group had significantly enhanced infiltration of CD86^+^ cells compared to the control and individual treatment groups (Fig. 8f and g). The proportion of CD8^+^ T cells among immune cells was significantly higher in the αPD-1+ES + AM group compared to the control and other treatment groups (Fig. 8h and i). The levels of various cytokines in the serum were measured to assess the immune response. αPD-1+ES + AM combination group had significantly higher levels of TNF-α, IL-6 and IL-1β compared to the Ctrl group (Fig. S34). Collectively, the combination of ES + AM and αPD-1 demonstrated the most effective tumor suppression and immune activation.

## 3 Conclusion

In conclusion, we developed a nanoparticle dyad for HCC immunotherapy comprising two functionally distinct modules. The E-SMSNα module, functionalized with anti-GPC3 antibodies, achieves targeted accumulation in HCC cells and delivers emodin to induce immunogenic cell death (ICD), thereby releasing tumor-associated antigens and damage-associated molecular patterns. The A-MSNm module, coated with DC membranes, homes to TDLNs and activates the STING pathway through c-di-AMP delivery, subsequently promoting DC maturation, antigen presentation, and CD8^+^ T cell priming. The E-SMSNα component exhibited potent tumoricidal activity both *in vitro* and *in vivo*, significantly reducing tumor growth and inducing apoptosis along with ICD-associated hallmarks. Concurrently, A-MSNm effectively induced DC maturation and antigen presentation, enhancing CD8^+^ T cell activation and promoting a robust adaptive immune response.

The combination of E-SMSNα and A-MSNm resulted in a robust immune response, as evidenced by increased infiltration of immune cells into the tumor microenvironment and elevated levels of pro-inflammatory cytokines. This multifaceted approach not only effectively suppressed primary tumor growth but also elicited a protective immune response against tumor rechallenge, as suggested by the expansion of memory T cell populations in spleens and TDLNs. Furthermore, the regimen synergized effectively with PD-1 immune checkpoint blockade, suggesting a promising combinatorial strategy for HCC immunotherapy.

The findings of this study demonstrate that nanoparticle-mediated immunotherapy can modulate the tumor microenvironment and enhance antitumor immunity in a subcutaneous HCC model. The developed nanoparticle dyad provides a multimodal strategy for overcoming local immune suppression, with potential for broader application in HCC immunotherapy.

## 4 Experimental section

### 4.1 Cell culture

The human hepatoma cell line HepG2, the mouse hepatoma cell line Hepa1-6 and human embryo kidney cell line 293T (HEK293T) were obtained from the Cell Bank of Chinese Academy of Sciences (Shanghai). Hepa1-6/OVA cells were purchased from Fuheng Cell Center (Shanghai, China). All cell lines were cultured in the Dulbecco's Modified Eagle Medium (DMEM), supplemented with 10% fetal bovine serum and 1% penicillin/streptomycin. Hepa1-6/luci cells were purchased from Fuheng Cell Center (Shanghai, China). Bone marrow cells sorted from C57BL/6 mice were cultured in the DMEM containing GM-CSF (20 ng/mL) and IL-4 (10 ng/mL) for 5 days to obtain BMDCs. Half of the medium was changed on the day 3. BMDCs were seeded in 24-well plates at a density of 1 × 10^6^ cells/well for treatments.

### 4.2 Preparation and characterization of E-SMSNα

The MSNs were synthesized as previously described [34,35]. Briefly, prepare a mixed water solution of CTAB and NaOH. The mass ratio of CTAB:NaOH:H_2_O is 1:0.05:100. Heat the mixture to 70°C and stir until the CTAB and NaOH are completely dissolved. Add a mixture of tetraethyl orthosilicate (TEOS) and cyclohexane to the CTAB/NaOH solution. The mass ratio of CTAB: NaOH: TEOS: cyclohexane: H_2_O is 1:0.05:7:23:100. Stir at 70°C and 950 rpm to form a stable microemulsion system for 54 h. The CTAB template was effectively removed through a triplicate ethanol washing process. For Emodin loading, 5 mL of CH_2_Cl_2_, 0.07 g of C_6_H_9_NO_2_, and 1.5 g of Emodin were sequentially added to 1.5 g of amino-modified MSN (spiky MSN, SMSN). The mixture was then heated to 60°C with 950 rpm for 3 h. For preparation of E-SMSNα, the anti-GPC3 antibody was conjugated to SMSN using 1-(3-dimethylaminopropyl)-3-ethylcarbodiimide hydrochloride (EDC)/N-hydroxysuccinimide (NHS) coupling procedure. Amino-modified SMSN was activated by 100 μL NHS (0.6 μg/mL) and 100 μL EDC (0.23 μg/mL) before incubating with anti-glypican-3 antibody (αGPC3) (Invitrogen, MA517083) for 2-4 h. The mixture was incubated with 10 μL αGPC3 for another 2-4 h, and was centrifuged at 5000 *g*, 4°C for 15 min to remove excessive EDC/NHS and unconjugated αGPC3.

### 4.3 Preparation and characterization of A-MSNm

For c-di-AMP loading onto MSN, 5 mL of anhydrous DMF, 0.2 g of c-di-AMP, and 2 g of MSN were sequentially added to a flask. The mixture was then sonicated for 30 min, followed by heating to 60°C with stirring at 800 rpm for 4 h. Afterward, the mixture was centrifuged at 3000 g for 20 min to separate A-MSN. To prepare BMDC membrane, femurs and tibias were taken out from male C57BL/6 mice aged 6-8 weeks, and the muscle tissue around the bones was removed with scissors. After both ends of the bones were excised, the marrow was extracted by syringe. The BMDCs were cultured in RPMI 1640 medium containing 10% FBS, penicillin, streptomycin, GM-CSF (20 ng/mL, TargetMol, TMPY-04114) and IL-4 (10 ng/mL, TargetMol, TMPY-02558). These BMDCs were then matured with LPS (10 μg/mL) for 24 h. The maturation status was verified by flow cytometry: cells were stained with anti-mouse CD11c-APC (BioLegend, 117310), anti-mouse CD80-PE/Cy7 (BioLegend, 104734), and anti-mouse CD86-APC/Cy7 (BioLegend, 105030), and analyzed using a FACSCelesta SORP (BD). Cell membranes were then extracted from the mature BMDCs as previously described [[35], [36], [37]]. Briefly, 1 × 10^8^ matured BMDCs were washed three times with PBS and ground in low permeability buffer containing EDTA-free protease inhibitors (Thermo Scientific, A32955). The nuclei were removed by centrifugation at 5000*g*, 4°C for 5 min. The supernatant was centrifuged at 150000 g, 4°C for 1 h. The cell membrane was collected and extruded 15 times through a 400 nm polycarbonate porous membrane with an Avanti mini-extruder (Avanti Research, 610020). For the cell membrane coating, 5 μL of the cell membrane solution (1 mg/mL) was added to the 100 μL A-MSN (1 mg/mL). The mixture was gently shaken at room temperature for 1 h to allow the cell membrane to adsorb onto the A-MSN surface. A-MSNm was then centrifuged at 5000 g for 15 min at 4°C to remove excess cell membrane. The final product was collected and resuspended in the desired buffer for further use.

To characterize the membrane protein composition, A-MSNm and native DC membranes were lysed and separated by SDS-PAGE. For Western blot analysis, proteins were transferred onto PVDF membranes and probed with primary antibodies: rabbit anti-MHC Class I (CST, 76828T), rabbit anti-CD80 (CST, 54521T), and rabbit anti-CD86 (CST, 19589T), followed by HRP-conjugated goat anti-rabbit IgG (CST, 7074S). Blots were developed using enhanced chemiluminescence and imaged.

### 4.4 Cell viability

The Cell viability of 293T cells was measured with the Cell Counting Kit-8 (CCK-8, Sigma-Aldrich). Cells were seeded in 96-well plate at a density of 5 × 10^3^ cells/well and incubated with E-SMSNα or A-MSNm (0-200 μg/mL). After washing with PBS, 10 μL of the CCK-8 solution was added to each well of the plate and incubated for 2 h at 37°C. The absorbance at 450 nm was detected with a microplate reader (Bio-Rad 680).

### 4.5 Targeted uptake of E-SMSNα

The HepG2 cells were incubated with E^FITC^-SMSN or E^FITC^-SMSNα for 90 min. The intensities of intracellular FITC signals were determined by imaging with confocal laser scanning microscope (Leica). To evaluate the targeting efficiency of E^FITC^-SMSNα *in vivo*, 2 × 10^6^ HepG2 cells suspended in 50 μL Matrigel were injected into the right posterior subcutaneous tissue of BALB/c-nu mice. When tumor volume reached approximately 100 mm^3^, E^FITC^ or E^FITC^-SMSNα was administered intravenously. Mice were then imaged using an *in vivo* imaging system (AniView100, Guangzhou Biolight Biotechnology Co.) at 6 h and 24 h post-injection to monitor FITC signals. Subsequently, to investigate the pharmacokinetic profile of Emo, mice were intravenously injected with either free Emo or Emo-loaded SMSNα. Serum samples were collected at 0, 12, 24, 36, and 48 h post-administration. The concentration of Emo in serum was quantified by measuring its characteristic absorption peak using ultraviolet-visible spectrophotometry.

### 4.6 Targeted efficiency of A-MSNm

Male C57BL/6 mice bearing Hepa1-6 tumors were included in this study. Cy7-MSN and Cy7-MSNm were administered via subcutaneous injection. After 24 h, the mice were euthanized, the major organs (heart, liver, spleen, lung, kidney, tumor and TDLN) were excised and imaged using a IVIS Spectrum imaging system (AniView100, Guangzhou Biolight Biotechnology Co., Ltd.).

### 4.7 *In vitro* cell apoptosis analysis

The annexin V assay was performed with the Cell Apoptosis Detection Kit (Beyotime, C1062S). In brief, the Hepa1-6 or HepG2 cells were incubated with SMSN, E-SMSN or E-SMSNα (100 μg/mL SMSN) for 24 h. Subsequently, the cells were collected, and approximately 1 × 10^6^ cells were resuspended. After centrifugation at 1000*g* for 5 min, the supernatant was discarded. The cell pellet was gently resuspended in 195 μL of Annexin V-FITC binding buffer. Then, 5 μL of Annexin V-FITC was added and mixed gently. Apoptosis was assessed by detecting FITC fluorescence using flow cytometry.

### 4.8 Immunogenic cell death (ICD) marker detection

Hepa1-6 cells were seeded in 24-well plates at a density of 3 × 10^4^ per well and treated with emodin or E-SMSNα for 24 h. For immunofluorescence staining, treated cells were fixed with 4% paraformaldehyde and incubated overnight at 4°C with primary antibodies against calreticulin (1:500, Immunoway, YM8162) and HMGB1 (1:500, ABclonal, A19529). After washing, cells were incubated with ABflo 488-conjugated goat anti-rabbit IgG (1:200, Abclonal, AS053) for CRT and AbFluor 594 goat anti-rabbit IgG (1:500, Immunoway, RS3611) for HMGB1. Nuclei were counterstained with DAPI, and images were captured using a fluorescence microscope (ZEISS Apotome 3). Extracellular ATP levels were measured using an ATP Assay Kit (Beyotime, S0026). Briefly, Hepa1-6 cells were treated as above, and the cell culture medium supernatants were collected. A 100 μL aliquot of supernatant was added to a black 96-well plate with a clear bottom, and luminescence was detected using a microplate reader (Cytation 5, BioTek).

### 4.9 DC maturation, antigen presentation, cross-presentation, and T cell activation assays

To evaluate STING pathway activation, BMDCs were treated with c-di-AMP or A-MSNm as indicated, and whole-cell lysates were subjected to Western blotting using rabbit anti-GAPDH (ABclonal, A19056), rabbit anti-STING/TMEM173 (ABclonal, A21051), rabbit anti-p-STING (CST, 72971S), rabbit anti-IRF3 (ABclonal, A19717), rabbit anti-p-IRF3 (CST, 29047S), and HRP-conjugated goat anti-rabbit IgG (CST, 7074S), signals were detected by enhanced chemiluminescence. Immunofluorescence staining of p-IRF3 also was performed.

To confirm that A-MSNm-mediated DC activation is STING-dependent, BMDCs were pretreated with C-176 (2 μM, MCE) for 2 h, followed by incubation with A-MSNm for 48 h. The cell culture supernatant was collected and IFN-β levels were quantified using a mouse IFN-β ELISA assay kit (Elabscience, E-EL-M0033). For gene expression analysis, total mRNA was extracted with TRIzol (Beyotime, R0016) and reverse-transcribed using ReverTra Ace® qPCR RT (TOYOBO, FSQ-101). Quantitative real-time PCR was performed on a Bio-Rad CFX96 Real-Time System using SYBR Green Mix (TOYOBO, QPK-201) with primers for *Irf7, Ifit1, Isg15, Oasl1,* and *Ccl5*. *Gapdh* was used as the internal control.

For DC maturation analysis, BMDC cells were incubated with A-MSNm for 48 h, and then stained with anti-mouse CD11c-APC (BioLegend, 117310), anti-mouse CD80-PE/Cy7 (BioLegend, 104733), and anti-mouse CD86-PE (BioLegend, 105007) for 30 min at 4°C. Gating strategy: CD11c^+^ cells were first gated, and the percentage of CD80^+^CD86^+^ cells within the CD11c^+^ population was quantified as mature DCs**.** The maturation status of BMDCs was evaluated using FACSCelesta SORP (BD).

For antigen presentation assessment, the A-MSNm treated BMDCs were pulsed with 1 μg/mL OVA_257-264_ peptide (InvivoGen, vac-sin) for 10 h, followed by detection of SIINFEKL-H-2Kb^+^ BMDCs using anti-SIINFEKL/H-2Kb antibody (BioLegend, 141607). To evaluate cross-presentation of tumor-derived antigens under more physiological conditions, Hepa1-6-OVA cells were first treated with E-SMSNα for 48 h to induce cell death. Subsequently, 1 × 10^6^ treated Hepa1-6-OVA cells were co-cultured with 2.5 × 10^5^ A-MSNm-activated BMDCs per well for 24 h. After removing non-engulfed tumor cells, BMDCs were collected and stained with CD11c-APC and SIINFEKL–H-2Kb antibody to quantify the percentage of SIINFEKL-H-2Kb^+^ BMDCs.

CD8^+^ T cells were isolated from spleens of euthanized OT-I mice aged 6–8 weeks. Spleens were gently dissociated in PBS using a syringe plunger, passed through 70 μm cell strainers, and treated with red blood cell lysis buffer (TIANGEN, RT122). Splenocytes were resuspended in autoMACS Running Buffer (Miltenyi, 130-091-221) and labeled with CD8a Microbeads (Miltenyi, 130-117-044). CD8^+^ T cells were isolated using an autoMACS® NEL Separator (Miltenyi) and then activated for 48 h in RPMI-1640 supplemented with anti-mouse CD3*ε* (5 μg/mL, BioLegend, 100302), anti-mouse CD28 (5 μg/mL, BioLegend, 102102), and IL-2 (100 U/mL, TargetMol, TMPY-02788). For CFSE labeling, CD8^+^ T cells were incubated with 5 μM CFSE (TargetMol, T6802) for 5 min at room temperature and washed with PBS containing 5% HIFCS.

For T cell activation, proliferation, and effector function analysis, BMDCs from either the peptide-pulsing or the cross-presentation assay were seeded in 96-well round-bottom plates at 1000 cells per well. CD8^+^ OT-I T cells (10,000 cells per well) were added at an effector-to-target ratio of 10:1. After 72 h of incubation, cells were harvested and stained with anti-mouse CD8a (BioLegend, 100708, 100714) and anti-mouse CD69-APC/Cy7 (BioLegend, 104525) to assess T cell activation. For intracellular cytokine staining, cells were fixed, permeabilized, and stained with anti-mouse IFN-γ-PE (BioLegend, 163503) and anti-human/mouse Granzyme B-APC (BioLegend, 372204). T cell proliferation was evaluated by CFSE dilution.

### 4.10 Animal treatment

Cultured Hepa1-6 tumor cells were trypsinized, centrifuged (800 rpm, 3 min), and washed twice in PBS. The subcutaneous liver cancer model was generated by injecting 2 × 10^6^ Hepa1-6 cells (in 50 μL matrigel) into the right posterior subcutaneous tissue of C57BL/6 mice (day 0). Tumor growth was monitored by a vernier caliper, and the tumor volume was calculated with the formula (volume = 0.5 × length × width [2]). E-SMSNα (100 μL, 50 mg/kg) was administered intravenously every 3 days, and A-MSNm (50 μL, 2 mg/kg) was administered via subcutaneous injection every 3 days. αPD-1 (5 mg/kg) was administered via intraperitoneal injection every 7 days. For tumor rechallenge studies, 2 × 10^6^ Hepa1-6/luci cells were injected subcutaneously into the right posterior subcutaneous tissue of C57BL/6 mice (day 0). E-SMSNα and A-MSNm were administered intravenously on day 5, 8, 11, 14 and 17. Surgical resection of the primary tumors was performed on day 18, and the incisions were carefully sutured. Tumor removal and potential recurrence were monitored using IVIS Spectrum imaging system (AniView100, Guangzhou Biolight Biotechnology Co., Ltd.) on indicated days. The cured mice and naïve controls were then received a secondary subcutaneous challenge of 2 × 10^6^ Hepa1-6/luci cells on day 48, and the tumor growth was subsequently monitored. On day 83, the spleens and tumor-draining lymph nodes (TDLNs) were harvested from euthanized mice, and the memory T cell populations were analyzed by FACS.

### 4.11 Quantification of serum cytokine levels by ELISA

To measure the levels of IFN-γ in mouse serum, 1 mL venous blood was harvested and centrifuged immediately at 3000 rpm for 20 min. The supernatant was collected and measured with the mouse IFN-γ ELISA assay kit (Beyotime, PI508). The levels of TNF-α and IL-6 in mouse serum were measured using the TNF-α ELISA kit (Boster, EK0527) and the IL-6 ELISA kit (Boster, EK0411) according to the manufacturer's instruction.

### 4.12 Flow cytometry

Tumors were homogenized in PBS to obtain single cell suspensions, incubated with appropriate antibodies for measurement on a BD FACSAria cell sorter, and analyzed by the FlowJo software (Tree Star). Zombie Aqua™ Fixable Viability Kit (BioLegend, 423101), anti-CD45-APC/Cy7 (eBioscience, 47045182), anti-mouse-CD45-FITC (BioLegend, 103108), anti-CD4-PE (Biolegend, 100408), anti-CD8-PerCP/Cy5.5 (BD, 551162), anti-human/mouse CD44-PE/Cy7 (BioLegend, 103030), anti-mouse CD62L-APC/CY7 (BioLegend, 104428), anti-mouse CD11c-APC (BioLegend, 117310), anti-mouse CD80-PE/Cy7 (BioLegend,104733), and anti-mouse CD86-PE (BioLegend, 105007), anti-mouse-CD86-APC/Cy7 (BioLegend, 105030), anti-mouse-CD40-PE (BioLegend, 157505), anti-mouse-CD197(CCR7)-PE/Cy7 (BioLegend, 120123), anti-mouse-IL12/IL-23 p40-PE/Cy7 (BioLegend, 505209), and anti-mouse-I/A/I-E (MHC-II)-APC/Cy7 (BioLegend, 107628) were used.

### 4.13 Statistical analysis

Statistical analysis was performed with GraphPad Prism 9.0. Data are shown as mean ± SD. For comparisons among three or more groups, one-way ANOVA with Tukey's post-hoc test was used. For tumor growth curves and other repeated-measurement datasets, two-way ANOVA was used. For comparisons between two groups, Student's t-test was used. For survival analysis, the Log-rank (Mantel–Cox) test was applied. Statistical significance was defined as *p < 0.05, **p < 0.01, ***p < 0.001.

## CRediT authorship contribution statement

**Chang Liu:** Conceptualization, Formal analysis, Funding acquisition, Writing – original draft. **Xiejun Zhao:** Methodology, Writing – original draft. **Hui Song:** Supervision. **Yiwen Yuan:** Software. **Jia Ma:** Project administration. **Yingtong Dong:** Visualization. **Tianzhao Xu:** Data curation. **Xiaojiao Li:** Writing – review & editing. **Xinghui Liu:** Resources.

## Declaration of competing interest

The authors declare that they have no known competing financial interests or personal relationships that could have appeared to influence the work reported in this paper.

## Data Availability

Data will be made available on request.

## References

[1] Singal A.G., Kanwal F., Llovet J.M. (2023). Global trends in hepatocellular carcinoma epidemiology: implications for screening, prevention and therapy. Nat. Rev. Clin. Oncol..

[2] Vogel A., Meyer T., Sapisochin G., Salem R., Saborowski A. (2022). Hepatocellular carcinoma. Lancet.

[3] Llovet J.M., Kelley R.K., Villanueva A., Singal A.G., Pikarsky E., Roayaie S., Lencioni R., Koike K., Zucman-Rossi J., Finn R.S. (2021). Hepatocellular carcinoma. Nat. Rev. Dis. Primers.

[4] Llovet J.M., Zucman-Rossi J., Pikarsky E., Sangro B., Schwartz M., Sherman M., Gores G. (2016). Hepatocellular carcinoma. Nat. Rev. Dis. Primers.

[5] Mazzaferro V., Sposito C., Zhou J., Pinna A.D., De Carlis L., Fan J., Cescon M., Di Sandro S., Yi-Feng H., Lauterio A., Bongini M., Cucchetti A. (2018). Metroticket 2.0 model for analysis of competing risks of death after liver transplantation for hepatocellular carcinoma. Gastroenterology.

[6] Wallace D., Cowling T.E., Walker K., Suddle A., Rowe I., Callaghan C., Gimson A., Bernal W., Heaton N., van der Meulen J. (2020). Short- and long-term mortality after liver transplantation in patients with and without hepatocellular carcinoma in the UK. Br. J. Surg..

[7] Sung H., Ferlay J., Siegel R.L., Laversanne M., Soerjomataram I., Jemal A., Bray F. (2021). Global cancer statistics 2020: GLOBOCAN estimates of incidence and mortality worldwide for 36 cancers in 185 countries. CA Cancer J. Clin..

[8] Tabrizian P., Abdelrahim M., Schwartz M. (2024). Immunotherapy and transplantation for hepatocellular carcinoma. J. Hepatol..

[9] Finn R.S., Ryoo B.Y., Hsu C.H., Li D., Burgoyne A.M., Cotter C., Badhrinarayanan S., Wang Y., Yin A., Edubilli T.R., Mahrus S., Secrest M.H., Shemesh C.S., Yu N., Hack S.P., Cha E., Gane E. (2025). Tiragolumab in combination with atezolizumab and bevacizumab in patients with unresectable, locally advanced or metastatic hepatocellular carcinoma (MORPHEUS-Liver): a randomised, open-label, phase 1b-2, study. Lancet Oncol..

[10] Sangro B., Sarobe P., Hervas-Stubbs S., Melero I. (2021). Advances in immunotherapy for hepatocellular carcinoma. Nat. Rev. Gastroenterol. Hepatol..

[11] Liu F., Liu Y., Chen Z. (2018). Tim-3 expression and its role in hepatocellular carcinoma. J. Hematol. Oncol..

[12] Yau T., Park J.W., Finn R.S., Cheng A.L., Mathurin P., Edeline J., Kudo M., Harding J.J., Merle P., Rosmorduc O., Wyrwicz L., Schott E., Choo S.P., Kelley R.K., Sieghart W., Assenat E., Zaucha R., Furuse J., Abou-Alfa G.K., El-Khoueiry A.B., Melero I., Begic D., Chen G., Neely J., Wisniewski T., Tschaika M., Sangro B. (2022). Nivolumab versus sorafenib in advanced hepatocellular carcinoma (CheckMate 459): a randomised, multicentre, open-label, phase 3 trial. Lancet Oncol..

[13] Rimassa L., Finn R.S., Sangro B. (2023). Combination immunotherapy for hepatocellular carcinoma. J. Hepatol..

[14] Ying S., Liu H., Zhang Y., Mei Y. (2025). Harnessing dendritic cell function in hepatocellular carcinoma: advances in immunotherapy and therapeutic strategies. Vaccines (Basel).

[15] Jeng L.B., Liao L.Y., Shih F.Y., Teng C.F. (2022). Dendritic-Cell-Vaccine-Based immunotherapy for hepatocellular carcinoma: clinical trials and recent preclinical studies. Cancers (Basel).

[16] Li W., Chen G., Peng H., Zhang Q., Nie D., Guo T., Zhu Y., Zhang Y., Lin M. (2024). Research progress on dendritic cells in hepatocellular carcinoma immune microenvironments. Biomolecules.

[17] European Association for the Study of the, L (2018). EASL Clinical Practice Guidelines: Management of hepatocellular carcinoma. J. Hepatol..

[18] Wei W.T., Lin S.Z., Liu D.L., Wang Z.H. (2013). The distinct mechanisms of the antitumor activity of emodin in different types of cancer. Oncol. Rep..

[19] Dong X., Fu J., Yin X., Cao S., Li X., Lin L., Huyiligeqi Ni J. (2016). Emodin: a review of its pharmacology, toxicity and pharmacokinetics. Phytother Res..

[20] Lu C., Wang W., Fu Y.X. (2026). Opportunities and challenges of targeting cGAS-STING in cancer. Nat. Rev. Cancer.

[21] Elmetwalli A. (2026). Ferroptosis and the cGAS-STING pathway into precision nano-immuno-theranostics: a mechanistic paradigm for reversing drug resistance in hepatocellular carcinoma. Drug Resist Updat.

[22] Ma Y.T., Zuo J., Kirkham A., Curbishley S., Blahova M., Rowe A.L., Bathurst C., Mehrzad H., Karkhanis S., Punia P., James M.W., Stern N., Rao A., Hull D., Lowe F., Sylla P., Webster L., Hussain S., Yap C., Palmer D., Adams D.H. (2025). Addition of dendritic cell vaccination to conditioning cyclophosphamide and chemoembolization in patients with hepatocellular carcinoma: the ImmunoTACE trial. Clin. Cancer Res..

[23] Gibhardt J., Commichau F.M. (2025). c-di-AMP is an envoy of inflammation. Nat. Chem. Biol..

[24] Wang D., Deng X., Wang J., Che S., Ma X., Zhang S., Dong Q., Huang C., Chen J., Shi C., Zhang M.R., Hu K., Luo L., Xiao Z. (2024). Environmentally responsive hydrogel promotes vascular normalization to enhance STING anti-tumor immunity. J Control Release.

[25] Haffner S.M., Parra-Ortiz E., Browning K.L., Jorgensen E., Skoda M.W.A., Montis C., Li X., Berti D., Zhao D., Malmsten M. (2021). Membrane interactions of virus-like mesoporous Silica nanoparticles. ACS Nano.

[26] Sang Z., Xu L., Ding R., Wang M., Yang X., Li X., Zhou B., Gou K., Han Y., Liu T., Chen X., Cheng Y., Yang H., Li H. (2023). Nanoparticles exhibiting virus-mimic surface topology for enhanced oral delivery. Nat. Commun..

[27] Liu M., Zhao Y., Shi Z., Zink J.I., Yu Q. (2023). Virus-like magnetic mesoporous Silica particles as a universal vaccination platform against pathogenic infections. ACS Nano.

[28] Shen D., Yang J., Li X., Zhou L., Zhang R., Li W., Chen L., Wang R., Zhang F., Zhao D. (2014). Biphase stratification approach to three-dimensional dendritic biodegradable mesoporous silica nanospheres. Nano Lett..

[29] Ding L., Zhu X., Wang Y., Shi B., Ling X., Chen H., Nan W., Barrett A., Guo Z., Tao W., Wu J., Shi X. (2017). Intracellular fate of nanoparticles with polydopamine surface engineering and a novel strategy for exocytosis-inhibiting, lysosome impairment-based cancer therapy. Nano Lett..

[30] Yang Z., Fan W., Zou J., Tang W., Li L., He L., Shen Z., Wang Z., Jacobson O., Aronova M.A., Rong P., Song J., Wang W., Chen X. (2019). Precision cancer theranostic platform by *in situ* polymerization in perylene diimide-hybridized hollow mesoporous organosilica nanoparticles. J. Am. Chem. Soc..

[31] Fu Y., Urban D.J., Nani R.R., Zhang Y.F., Li N., Fu H., Shah H., Gorka A.P., Guha R., Chen L., Hall M.D., Schnermann M.J., Ho M. (2019). Glypican-3-Specific antibody drug conjugates targeting hepatocellular carcinoma. Hepatology.

[32] Hassan H.M., Hamdan A.M., Alattar A., Alshaman R., Bahattab O., Al-Gayyar M.M.H. (2024). Evaluating anticancer activity of emodin by enhancing antioxidant activities and affecting PKC/ADAMTS4 pathway in thioacetamide-induced hepatocellular carcinoma in rats. Redox Rep..

[33] Zhang Q., Chen W.W., Sun X., Qian D., Tang D.D., Zhang L.L., Li M.Y., Wang L.Y., Wu C.J., Peng W. (2022). The versatile emodin: a natural easily acquired anthraquinone possesses promising anticancer properties against a variety of cancers. Int. J. Biol. Sci..

[34] Zhang Q., Dehaini D., Zhang Y., Zhou J., Chen X., Zhang L., Fang R.H., Gao W., Zhang L. (2018). Neutrophil membrane-coated nanoparticles inhibit synovial inflammation and alleviate joint damage in inflammatory arthritis. Nat. Nanotechnol..

[35] Wang Z., Zhang F., Shao D., Chang Z., Wang L., Hu H., Zheng X., Li X., Chen F., Tu Z., Li M., Sun W., Chen L., Dong W. (2019). Janus nanobullets combine photodynamic therapy and magnetic hyperthermia to potentiate synergetic anti-metastatic immunotherapy. Adv. Sci..

[36] Shen Y., Guo D., Ji X., Zhou Y., Liu S., Huang J., Song H. (2022). Homotypic targeting of immunomodulatory nanoparticles for enhanced peripheral and central immunity. Cell Prolif..

[37] Liu C., Zhou Y., Guo D., Huang Y., Ji X., Li Q., Chen N., Fan C., Song H. (2023). Reshaping intratumoral mononuclear phagocytes with antibody-opsonized immunometabolic nanoparticles. Adv. Sci. (Weinh.).

