# Supplementary material for: Synergistic nanoparticle dyad improving tumor immunogenicity and reshaping tolerogenic dendritic cells in hepatocellular carcinoma
Source: Mater Today Bio. 2026 Jul 23;39:103488. doi: 10.1016/j.mtbio.2026.103488 (PMC13446102; doi:10.1016/j.mtbio.2026.103488)
Supplement: Supplementary file 1 [file mmc1.docx]

Supporting Information

**Synergistic Nanoparticle Dyad Improving Tumor Immunogenicity and Reshaping Tolerogenic Dendritic Cells in Hepatocellular Carcinoma**

Liu Chang^1,^ †, Zhao Xiejun^2,^ †, Song hui^1,^ †, Yuan Yiwen^1^, Ma Jia^2,^, Dong Yingtong^3^, Xu Tianzhao^4,^ *, Li Xiaojiao^3,^ *, Liu Xinghui^1,^ *.

*1 Department of Clinical Laboratory, Pudong Gongli Hospital, Shanghai University of Medicine&Health Sciences, Shanghai, China*

*2* *School of Pharmacy, Health Science Center, Xi’an Jiaotong University, Xi'an, Shaanxi, PR China*

*3* *BioBank, The First Affiliated Hospital of Xi'an Jiaotong University, Xi'an 710061, Shaanxi, PR China.*

*4 Hospital Department, Shanghai University of Medicine and Health Sciences Affiliated to Zhoupu Hospital, Shanghai, China*

* Corresponding author: syliuxh@163.com; *lixiaojiao@xjtu.edu.cn; tianzhaoxufan@163.com*

† These authors contributed equally.

This supplemental file includes:

Figs. S1 to S34

**Figure S1.** The images of unloaded SMSN, fluorescent antibody-coated SMSN (SMSNα) and fluorescent antibody under visible light or 405 nm laser irradiation.

**Figure S2.** Absorbance characteristics of Emo at different concentrations. (a) UV-Vis absorption spectra of Emo at concentrations of 0, 1.25, 2.5, and 5 mg/mL. Characteristic absorption peak of Emo showed at 440 nm. (b) Calibration curve illustrating the linear relationship between Emo concentration (mg/mL) and absorbance at 440 nm. The equation of the line is Y=11.13X−0.7346 with a high coefficient of determination (R^2^=0.995), indicating a strong linear correlation.

**Figure S3**. Drug loading and release profiles of E-SMSNα and A-MSNm. (a) Loading capacity of Emoin E-SMSNα at varying weight ratios. (b) Cumulative release profile of Emo from E-SMSNα over 72 h under different pH conditions. (c) Loading capacity of c-di-AMP in A-MSNm at varying weight ratios. (d) Cumulative release profile of c-di-AMP from A-MSNm under different pH conditions. Data are represented as mean ±SD (n=3).

**Figure S4.** EDS elemental mapping of A-MSNm composed of oxygen (O), nitrogen (N), silicon (Si), phosphorus (P), and sulfur (S). Scale bar =50 nm.

**Figure S5.** (a) Hydrodynamic size distribution of unloaded MSN, A-MSN and A-MSNm. (b) Zeta potential of MSN, A-MSN and A-MSNm. (c) Size of A-MSNm in serum stored for 24h. (d) Zeta potential of A-MSNm in serum stored for 24h. Data are represented as mean ±SD (n=3). Statistical significance was calculated via a two-tailed Student's *t*-test. ns means no significance.

**Figure S6.** The cytotoxicity of E-SMSNα at different concentrations (0, 10, 50, 100, 150, 200 *μ*g/mL) was measured after 24-hour incubation in 293T cells. Data are represented as mean ±SD (n=3). One-way ANOVA with Tukey’s post-hoc test, ****p*<0.001.

**Figure S7.** The cytotoxicity of A-MSNm at different concentrations (0, 10, 50, 100, 150, 200 *μ*g/mL) was measured after 24-hour incubation in 293T cells. Data are represented as mean ±SD (n=3).

**Figure S8.** Cell viability was examined at indicated time points after incubation with 100 *μ*g/mL E-SMSNα in 293T cells. Data are represented as mean ±SD (n=3).

**Figure S9.** Cell viability was examined at indicated time points after incubation with 100 *μ*g/mL A-MSNm in 293T cells. Data are represented as mean ±SD (n=3).

**Figure S10.** Targeting efficiency of E-SMSNα *in vitro.* (a) Relative *GPC3* levels in 293T, THP1, HepG2, Hep3B, Huh7 cells and Hepa1-6. (b) Representative fluorescent images of E^FITC^-SMSN and E^FITC^SMSNα in HepG2 cells at 40 or 90 minutes. (c) Quantification of E^FITC^-SMSN and E^FITC^-SMSNα fluorescence intensity of HepG2 cells at 40 or 90 minutes. Data are represented as mean ±SD. One-way ANOVA with Tukey’s post-hoc test, ****p*<0.001.

**Figure S11.** Targeting efficiency of E-SMSNα *in vitro.* (a) Representative fluorescent images of E-SMSN, E-SMSNα and E-SMSNα+αGPC3(anti-glypican-3 antibody) in Hepa1-6 cells at 40 or 90 minutes. (b) Quantification of E-SMSN, E-SMSNα and E-SMSNα+αGPC3 in Hepa1-6 cells at 40 or 90 minutes. Data are represented as mean ±SD. One-way ANOVA with Tukey’s post-hoc test, **p*<0.01, ****p*<0.001, ns, not significant.

**Figure S12.** Targeting efficiency of E-SMSNα *in vivo.* (a) *In vivo* fluorescence imaging of HepG2 tumor bearing mice at different time points after FITC labeled E-SMSNα injection. (b) Emo and E-SMSNα concentrations in mouse serum over time. Data are represented as mean ±SD (n=3).

**Figure S13.** *In vivo* fluorescence imaging of Hepa1-6 tumor bearing mice at different time points after FITC labeled E-SMSN or E-SMSNα injection.

**Figure S14.** H&E staining on major organs collected from Hepa1-6 tumor-bearing mice 20 days after E-SMSNα and A-SMSNm tail vein injection. Scale bars = 50 *μ*m.

**Figure S15.** Biodistribution analysis reveals enhanced TDLN accumulation of A-MSNm. (a) *Ex vivo* fluorescence imaging of major organs (heart, liver, spleen, lung, kidney), tumors, and TDLNs from Hepa1-6 tumor-bearing mice 24 h after subcutaneous injection of Cy7-MSN or Cy7-MSNm. (b) Quantification of average fluorescence signal intensity in each tissue. Data are mean ± SD (n = 3). Statistical significance was calculated via a two-tailed Student's *t*-test. ns means no significance,****p* < 0.001.

**Figure S16**. Biochemical and hematological safety assessment of the nanoparticle dyad in Hepa1-6 tumor-bearing mice. Serum levels of (a) alkaline phosphatase (ALP) and (b) alanine aminotransferase (ALT) are expressed as percentages relative to normal reference values. Complete blood count parameters include (c) platelets (PLT), (d) red blood cells (RBC), and (e) white blood cells (WBC). Data are represented as mean ± SD (n = 3). ns, not significant.

**Figure S17** Annexin V^+^ cells quantification. Data are represented as mean ±SD (n=3). One-way ANOVA with Tukey’s post-hoc test, ****p*<0.001.

**Figure S18.** Apoptosis induction by E-SMSNα in Hepa1-6 cells. (a) Representative flow cytometry histograms of Annexin V staining in Hepa1-6 cells treated with different groups for 24 h. (b) Quantification of Annexin V⁺ cells across treatment groups. Data are represented as mean ± SD (n = 3). One-way ANOVA with Tukey's post-hoc test, ***p* < 0.01, ****p* < 0.001.

**Figure S19**. Inhibition of Hepa1-6 cell invasion and migration by E-SMSNα. (a) The effects of E-SMSNα on cell invasion. (b) Quantification of (a). (c) The effects of E-SMSNα on cell migration. The contour of cell migration was labeled by dashed lines. (d) Quantification of (c). Data are represented as mean ±SD (n=3). One-way ANOVA with Tukey’s post-hoc test, ***p*<0.01, ****p*<0.001.

**Figure S20**. E-SMSNα induces immunogenic cell death (ICD) in Hepa1-6 cells. (a) Immunofluorescence staining of CRT (green) in Hepa1-6 cells treated with control (Ctrl), Emodin (Emo), or E-SMSNα. (b) Immunofluorescence staining of HMGB1 (red) in different groups. (c) Quantification of extracellular ATP levels in the supernatant. Data are mean ± SD (n = 3). Statistical significance was calculated via one-way ANOVA with Tukey's multiple-comparison post-hoc test. **p* < 0.05, ***p* < 0.01. Scale bar: 100 μm.

**Figure S21**. Western blot analysis of STING pathway activation by A-MSNm in BMDCs.

**Figure S22.** Immunofluorescence staining analysis illustrating the effects of various treatments on the protein levels of p-IRF3. Scale bars =20 *μ*m.

**Figure S23.** A-MSNm-induced BMDC activation is mediated by the STING pathway. (a) Secreted IFN-β protein levels in BMDC supernatants treated with MSNm, A-MSNm, or A-MSNm+C176. (b) Relative mRNA expression of STING downstream ISGs in BMDCs was assessed by qPCR. Data are mean ± SD (n = 3). Statistical significance was calculated by One-way ANOVA with Tukey’s post-hoc test, **p* < 0.05, ***p* < 0.01, ****p* < 0.001.

**Figure S24.** Gating strategy for flow cytometric analysis of BMDC maturation and antigen-specific T cell responses *in vitro*. Representative gating hierarchies used to identify (a) mature CD11c⁺CD80⁺CD86⁺ BMDCs, (b) CD11c⁺SIINFEKL–H-2Kb⁺ BMDCs, (c) proliferating CFSE-labeled OT-I CD8⁺ T cells, (d) CD69⁺CD8⁺ T cells, (e) IFN-γ⁺CD8⁺ T cells, and (f) Granzyme B⁺CD8⁺ T cells. All analyses were performed on single cells gated from forward/side scatter (FSC/SSC) plots. Percentages indicate the proportion of parent population within each gate.

**Figure S25.** A-MSNm enhances the cross-presentation of tumor-derived antigens in vitro. (a) Schematic illustration of the in vitro cross-presentation assay. BMDCs were pre-treated with or without A-MSNm, followed by co-culture with E-SMSNα-treated Hepa1-6-OVA tumor cells for 24 h. After removing un-engulfed tumor cells, BMDCs were collected, isolated, and co-cultured with OT-I CD8^+^ T cells. (b) Representative flow cytometry plots and quantitative analysis of SIINFEKL-H-2Kb^+^ BMDC proportions in BMDCs. (c) Representative flow cytometry plots and quantitative analysis of CD69 expression in OT-I CD8^+^T cells. . Data are mean ± SD (n = 5). Statistical significance was calculated via a two-tailed Student’s *t*-test. ****p* < 0.001.

**Figure S26.** A-MSNm reprograms tolerogenic dendritic cells toward an immunostimulatory phenotype in vivo. (a) Representative flow cytometric images and quantification of CD80⁺CD86⁺ DCs (gated on CD45⁺CD11c⁺ cells) in TDLNs of Hepa1-6 tumor-bearing mice treated with PBS or A-MSNm. (b) MHC II⁺CD40⁺ DCs in TDLNs. (c) CCR7⁺ DCs in TDLNs. (d) MHC II⁺CD40⁺ DCs in tumor tissues. (e) IL-12⁺ DCs in tumor tissues. Complete gating strategies for flow cytometric analyses are provided in Figure S27-28. Data are mean ± SD (n = 5). Statistical significance was calculated via a two-tailed Student’s *t*-test. ****p < 0.001.*

**Figure S27.** Gating strategy for the identification of immunostimulatory and migratory DCs in tumor-draining lymph nodes (TDLNs). Representative gating hierarchies used to identify and characterize DCs. (a-c) Singlets were selected by FSC/SSC, followed by exclusion of Zombie-BV421^+^ cells and gating on CD45⁺ leukocytes. CD11c⁺ DCs were then identified for analysis of (a) CD80/CD86, (b) MHC-II/CD40, and (c) CCR7 expression. Percentages indicate the proportion of the parent population within each gate.

**Figure S28.** Gating strategy for identifying functional DCs within the immunosuppressive tumor microenvironment. Singlets were selected based on FSC/SSC properties, followed by exclusion of Zombie-BV421^+^ dead cells and gating on CD45^+^ leukocytes. CD11c^+^ DCs were then identified for analysis of (a) MHC-II/CD40 and (b) IL-12 expression. Percentages indicate the proportion of the parent population within each gate.

**Figure S29. The nanoparticle Dyad enhances DC activation within the tumor microenvironment.** (a,b) Quantification of CD86^+^ (a) and CD80^+^ (c) cells among CD11c⁺ DCs across treatment groups. (c,d) Representative gating strategy for CD11c^+^CD86^+^ DCs (c) and CD11c^+^CD80^+^ DCs (d). Data are represented as mean ±SD (n=3). One-way ANOVA with Tukey’s post-hoc test, ***p*<0.01, ****p*<0.001.

**Figure S30.** (a) Representative fluorescence microscopy images showing DAPI (blue) and CD86 (red) staining in different treatment groups: Ctrl, A-MSNm, E-SMSNα, and E-SMSNα+A-MSNm. Scale bar=100 *μ*m. (b) Quantification of CD86 fluorescence intensity. Data are represented as mean ±SD. One-way ANOVA with Tukey’s post-hoc test, ***p*<0.01, ****p*<0.001.

**Figure S31.** The proportion of CD8^+^ T cells from different treatment groups infiltrating into the tumor microenvironment. Flow cytometric analysis of the percentage of CD8^+^ T cells population in immune cells (gated on CD45^+^) (n = 3). One-way ANOVA with Tukey’s post-hoc test, **p*<0.05, ***p*<0.01, ****p*<0.001.

**Figure S32.** Cytokine Levels in Hepa1-6 HCC-bearing mice. (a) TNF-α, (b) IL-6, (c) IL-1β levels by different treatment. Data are represented as mean ±SD (n=3). One-way ANOVA with Tukey’s post-hoc test, **p*<0.05, ***p*<0.01, ****p*<0.001.

**Figure S33.** Gating strategy for identifying CD8⁺ Tcm and CD8⁺ Tem cells in spleens (a) and TDLN (b). Singlets were selected based on FSC/SSC properties, followed by exclusion of Zombie-BV421⁺ dead cells and gating on CD45⁺ leukocytes. CD3⁺CD8⁺ T cells were identified, and and memory subsets were defined as Tcm (CD44⁺CD62L⁺) or Tem (CD44⁺CD62L⁻). Percentages indicate the proportion of the parent population within each gate.

**Figure S34.** Cytokine Levels in Hepa1-6 HCC-bearing mice. (a) TNF-α, (b) IL-6, (c) IL-1β levels by different treatment. Data are represented as mean ±SD (n=3). One-way ANOVA with Tukey’s post-hoc test, **p*<0.05, ****p*<0.001.
